# Detection of Cistanches Herba (*Rou Cong Rong*) Medicinal Products Using Species-Specific Nucleotide Signatures

**DOI:** 10.3389/fpls.2018.01643

**Published:** 2018-11-13

**Authors:** Xiao-yue Wang, Rong Xu, Jun Chen, Jing-yuan Song, Steven-G Newmaster, Jian-ping Han, Zheng Zhang, Shi-lin Chen

**Affiliations:** ^1^Key Laboratory of Bioactive Substances and Resources Utilization of Chinese Herbal Medicine, Ministry of Education, Institute of Medicinal Plant Development, Chinese Academy of Medicinal Science and Peking Union Medicinal College, Beijing, China; ^2^NHP Research Alliance, Biodiversity Institute of Ontario (BIO), University of Guelph, Guelph, ON, Canada; ^3^Key Laboratory of Beijing for Identification and Safety Evaluation of Chinese Medicine, Institute of Chinese Materia Medica, China Academy of Chinese Medical Sciences, Beijing, China

**Keywords:** Cistanches Herba, Chinese patent medicine, nucleotide signature, degraded DNA, medicine quality control

## Abstract

Cistanches Herba is a medicinal plant that has tonification properties and is commonly used in Asia. Owing to the imbalance between supply and demand, adulterants are frequently added for profit. However, there is no regulatory oversight because quality control tools are not sufficient for identifying heavily processed products. Thus, a novel molecular tool based on nucleotide signatures and species-specific primers was developed. The ITS2 regions from 251 Cistanches Herba and adulterant samples were sequenced. On the basis of SNP sites, four nucleotide signatures within 30~37 bp and six species-specific primers were developed, and they were validated by artificial experimental mixtures consisting of six different species and different ratios. This method was also applied to detect 66 Cistanches Herba products on the market, including extracts and Chinese patent medicines. The results demonstrated the utility of nucleotide signatures in identifying adulterants in mixtures. The market study revealed 36.4% adulteration: 19.7% involved adulteration with *Cynomorium songaricum* or *Cistanche sinensis*, and 16.7% involved substitution with *Cy. songaricum, Ci. sinensis*, or *Boschniakia rossica*. The results also revealed that *Cy. songaricum* was the most common adulterant in the market. Thus, we recommend the use of species-specific nucleotide signatures for regulating adulteration and verifying the quality assurance of medicinal product supply chains, especially for processed products whose DNA is degraded.

## Introduction

Cistanches Herba (*Rou Cong Rong*) is a well-known Pharmacopoeia-recorded medicine in Asia (Chinese Pharmacopoeia Commission, 2015; Japan Pharmacopeial Convention, 2016); this medicine is derived from the dried succulent stems of *Cistanche deserticola* Y. C. Ma or *Cistanche tubulosa* (Schenk) Wight. Cistanches Herba has been used for more than 3,000 years as a superior tonic, as it is not toxic and can be taken for long periods of time (Li et al., 2016). Furthermore, Cistanches Herba was bestowed with the honor of being named “Desert Ginseng” because of its great medicinal value, especially in strengthening male sexual function (Zhang and Su, 2014; Gu et al., 2016). There are more than 100 Chinese patent medicines recorded in the Chinese Pharmacopoeia Commission (2015) and in other local official promulgated standards (Wang et al., 2012). As the population of elderly individuals increases, there is considerable demand for Cistanches Herba and its medicinal products. However, raw material resources are becoming increasingly scarce. In fact, the two original species of Cistanches Herba have been added to the China Plant Red Data Book as state-protected wild plants (category II) (Fu, 1991). The medicinal materials on the market are mainly cultivated in northwestern China.

Owing to the considerable imbalance between the supply and demand of Cistanches Herba, many adulterants have entered the market; these adulterants are inconsistent with standards and can threaten drug security. The known adulterants include the dried succulent stems of *Cynomorium songaricum* Rupr. (Cynomorii Herba, *Suo Yang* in Chinese), *Cistanche sinensis* Beck, *Orobanche coerulescens* Stephan, and *Boschniakia rossica* (Cham. et Schlecht.) Fedtsch. et Flerov (Sun et al., 2012). These adulterants have morphological characteristics similar to those of Cistanches Herba, making traditional taxonomic identification difficult, particularly after the material is processed into medicinal products. Microscopic identification is not available because Cistanches Herba has no definitive or unique microscopic characteristics. Current analytical chemistry tools are not sufficient for detecting adulteration of Cistanches Herba because similar compounds also exist within the known adulterants *Ci. salsa* (Lei et al., 2001; Chen et al., 2007) and *Ci. sinensis* (Liu et al., 2013). Therefore, the development of a rapid molecular method for the authentication of Cistanches Herba and its products is urgently needed for proper quality control systems in Chinese patent medicine and other medicinal product industries.

Chen et al. first suggested internal transcribed spacer 2 (ITS2) as a universal barcode for medicinal plants (Chen et al., 2010). Sun et al. verified the ITS2 region as a preferable DNA barcode for identifying Cistanches Herba and its adulterants (Sun et al., 2012). However, ITS2 cannot be used to distinguish Chinese patent medicine with degraded DNA. Recently, an increasing number of studies have shown that the “mini barcode” is a useful method for amplifying degraded DNA (Hajibabaei et al., 2006; Meusnier et al., 2008; Dubey et al., 2011; Lo et al., 2015). However, nucleotide signatures are more appropriate than mini barcodes, as the formers refer to one or more nucleotides that are unique to one species and can be effectively utilized by many molecular techniques, such as DNA probes, microfluidics and loop-mediated isothermal amplification (de Boer et al., 2015). Han et al. developed nucleotide signatures for *Panax ginseng, Angelica sinensis* and *Lonicera japonica* and successfully identified the associated Chinese patent medicines (Liu et al., 2016; Wang et al., 2016a; Gao et al., 2017).

The goal of our research focused on the development of nucleotide signatures for the identification of adulterants in functional products containing Cistanches Herba. Specifically, we (1) developed four nucleotide signatures and six specific primer pairs for differentiating authentic Cistanches Herba and known adulterants, (2) validated the four nucleotide signatures in experiments including mixtures of known taxonomic vouchers used to prepare Cistanches Herba products that contain both authentic and adulterated ingredients, and (3) performed a market survey of 66 Cistanches Herba products via their nucleotide signatures.

## Materials and methods

### Sample collection and preparation

**Figure 1:**
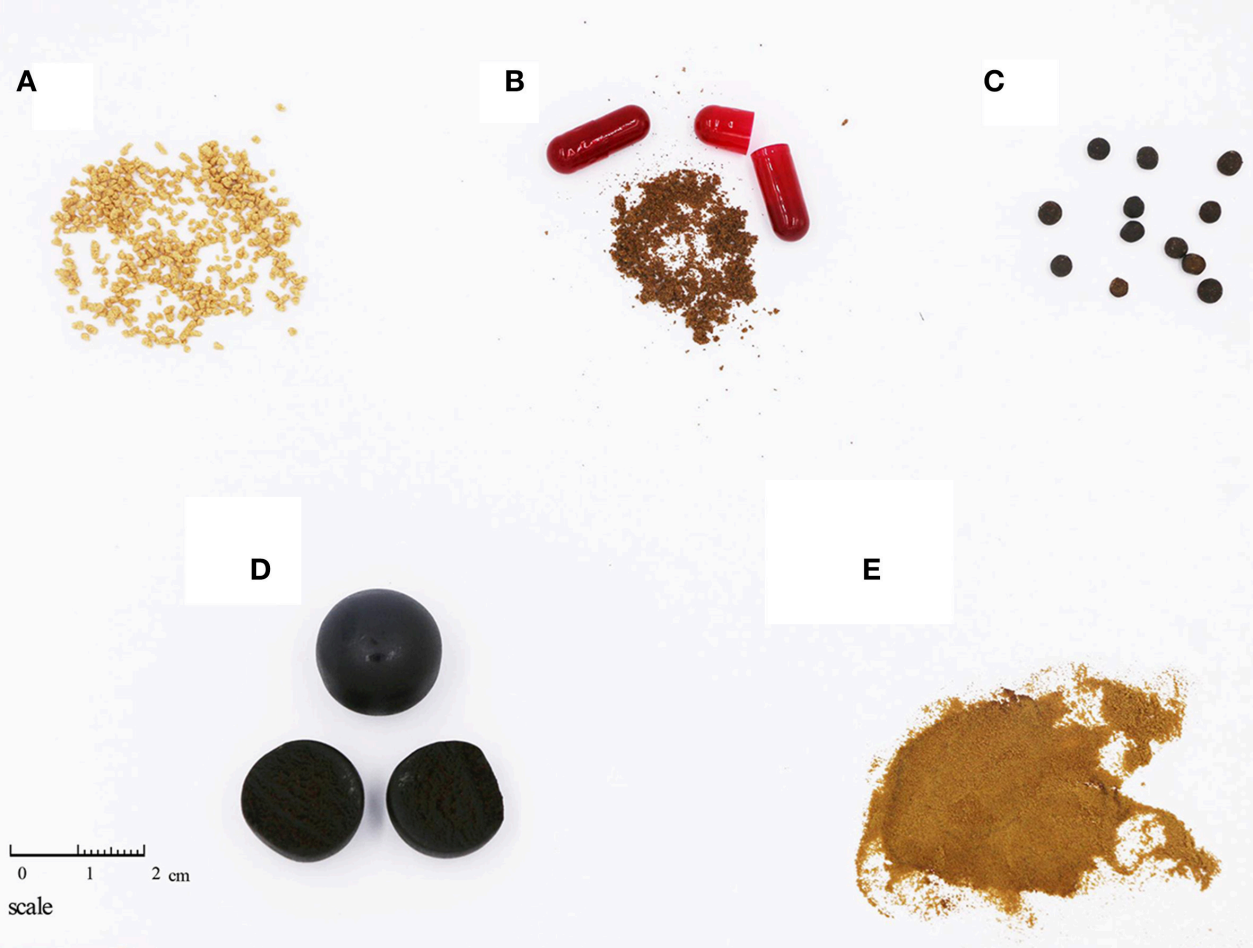
Different morphological characteristics of different dose forms of Cistanches Herba products. **(A)** Particles (ZCY29, Wenweishu particles); **(B)** Capsules (ZCY35, Sanbao capsules); **(C)** Water honeyed pills (ZCY70, Shihu Yeguang pills); **(D)** Large honeyed pills (ZCY48, Shihu Yeguang pills); **(E)** Extracts (YC23).

In total, 251 samples were collected from Inner Mongolia, Xinjiang, and Ningxia, among other areas; these samples included 214 Cistanches Herba and 37 adulterants and are detailed in Supplementary Table S1. Corresponding voucher samples were validated by taxonomists and deposited in the Herbarium of the Institute of Medicinal Plant Development, Chinese Academy of Medical Sciences, Beijing, China. A total of 35 batches of powders, slices and extracts of Cistanches Herba were purchased from online stores and brick-and-mortar drugstores in Beijing and Chengdu (Table 1). In total, 31 batches of Chinese patent medicine containing Cistanches Herba were purchased from different drugstores (Table 2), and the declared compositions of different Chinese patent medicines are shown in Supplementary Table S3. The different morphological characteristics of different dose forms are shown in Figure 1.

**Table 1:** Sample information and identification results of the 35 powders, medicinal slices, and extract.

**Sample No**.	**Latin name of medicinal materials**	**Sample type**	**Collection site**	**Collection approach**	**Identification result**
YC01	Cistanches Herba	Medicinal slices	Beijing	Offline	*Cynomorium songaricum*
YC05	Cistanches Herba	Medicinal slices	Chengdu, Sichuan	Offline	*Cistanche deserticola*
YC06	Cistanches Herba	Medicinal slices	Chengdu, Sichuan	Offline	*Cistanche tubulosa*
YC09	Cistanches Herba	Medicinal slices	Chengdu, Sichuan	Offline	*Cistanche deserticola*
YC11	Cistanches Herba	Medicinal slices	Chengdu, Sichuan	Offline	*Cistanche tubulosa*
YC13	Cistanches Herba	Medicinal slices	Chengdu, Sichuan	Offline	*Salvia miltiorrhiza*
YC17	Cistanches Herba	Medicinal slices	Chengdu, Sichuan	Offline	*Cistanche tubulosa*
YC24	Cistanches Herba	Medicinal slices	Anhui Bozhou Herb Market	Offline	*Cistanche deserticola*
YC25	Cistanches Herba	Medicinal slices	Anhui Bozhou Herb Market	Offline	*Cistanche deserticola*
YC26	Cistanches Herba	Medicinal slices	Anhui Bozhou Herb Market	Offline	*Cistanche tubulosa*
YC27	Cistanches Herba	Medicinal slices	Anhui Bozhou Herb Market	Offline	*Cistanche deserticola*
YC28	Cistanches Herba	Medicinal slices	Anhui Bozhou Herb Market	Offline	*Cistanche deserticola*
YC29	Cistanches Herba	Medicinal slices	Anhui Bozhou Herb Market	Offline	*Cistanche deserticola*
YC30	Cistanches Herba	Medicinal slices	Anhui Bozhou Herb Market	Offline	*Cistanche tubulosa*
YC31	Cistanches Herba	Medicinal slices	Anhui Bozhou Herb Market	Offline	*Cistanche deserticola*
YC32	Cistanches Herba	Medicinal slices	Anhui Bozhou Herb Market	Offline	*Cistanche deserticola*
YC33	Cistanches Herba	Medicinal slices	Anhui Bozhou Herb Market	Offline	*Cistanche tubulosa*
YC34	Cistanches Herba	Medicinal slices	Anhui Bozhou Herb Market	Offline	*Cistanche deserticola*
YC35	Cistanches Herba	Medicinal slices	Anhui Bozhou Herb Market	Offline	*Cistanche deserticola*
YC36	Cistanches Herba	Medicinal slices	Anhui Bozhou Herb Market	Offline	*Cistanche deserticola*
YC37	Cistanches Herba	Medicinal slices	Anhui Bozhou Herb Market	Offline	*Cistanche deserticola*
YC38	Cistanches Herba	Medicinal slices	Anhui Bozhou Herb Market	Offline	*Cistanche deserticola*
YC39	Cistanches Herba	Medicinal slices	Anhui Bozhou Herb Market	Offline	*Cistanche tubulosa*
YC40	Cistanches Herba	Medicinal slices	Anhui Bozhou Herb Market	Offline	*Cistanche tubulosa*
YC41	Cistanches Herba	Medicinal slices	Anhui Bozhou Herb Market	Offline	*Cistanche tubulosa*
YC42	Cistanches Herba	Medicinal slices	Anhui Bozhou Herb Market	Offline	*Cistanche tubulosa*
YC43	Cistanches Herba	Medicinal slices	Anhui Bozhou Herb Market	Offline	*Cistanche tubulosa*
YC03	Cistanches Herba	Powder	Anhui Bozhou Herb Market	Online	*Cynomorium songaricum*
YC04	Cistanches Herba	Powder	Anhui Bozhou Herb Market	Online	*Cistanche deserticola, Cistanche tubulosa, Cynomorium songaricum, Cistanche sinensis*
YC18	Cistanches Herba	Powder	Anhui Bozhou Herb Market	Online	*Cistanche deserticola, Cistanche tubulosa, Cynomorium songaricum, Cistanche sinensis*
YC19	Cistanches Herba	Powder	Anhui Bozhou Herb Market	Online	*Cistanche tubulosa, Cynomorium songaricum, Cistanche sinensis*
YC20	Cistanches Herba	Powder	Anhui Bozhou Herb Market	Online	*Cistanche deserticola, Cistanche tubulosa, Cynomorium songaricum*
YC21	Cistanches Herba	Powder	Anhui Bozhou Herb Market	Online	*Cistanche deserticola, Cistanche tubulosa, Cynomorium songaricum, Cistanche sinensis*
YC22	Cistanches Herba	Powder	Anhui Bozhou Herb Market	Online	*Cistanche sinensis, Cistanche tubulosa, Cynomorium songaricum*
YC23	Cistanches Herba	Extract	Dalian, Liaoning	Online	*Cistanche sinensis*

**Table 2:** Identification results of Chinese patent medicines based on the nucleotide signatures.

**Sample no**.	**Sample name**	**Collection site**	**Collection approach**	**Identification results of the related species**
ZCY16	Shihu Yeguang pills	Beijing	Offline	*Cynomorium songaricum, Cistanche deserticola*
ZCY26	Shihu Yeguang pills	Beijing	Offline	*Cynomorium songaricum*
ZCY29	Wenweishu particles	Shanghai	Online	*Cynomorium songaricum*
ZCY33	Shihu Yeguang pills	Taiyuan, Shanxi	Online	*Cynomorium songaricum, Cistanche tubulosa*
ZCY34	Shihu Yeguang pills	Shaoguan, Guangdong	Online	*Cistanche tubulosa*
ZCY35	Sanbao capsules	Shaoguan, Guangdong	Online	*Cynomorium songaricum, Cistanche deserticola, Cistanche sinensis*
ZCY40	Kangguzhi Zengsheng pills	Shaoguan, Guangdong	Online	*Cynomorium songaricum*
ZCY41	Kanggu Zengsheng pills	Shaoguan, Guangdong	Online	*Cynomorium songaricum, Cistanche tubulosa*
ZCY44	Shihu Yeguang pills	Changsha, Hunan	Online	*Cynomorium songaricum*
ZCY48	Shihu Yeguang pills	Beijing	Online	*Cynomorium songaricum*
ZCY51	Shihu Yeguang pills	Dezhou, Shandong	Offline	*Cistanche tubulosa*
ZCY53	Shihu Yeguang pills	Dezhou, Shandong	Offline	*Cistanche deserticola*
ZCY55	Shihu Yeguang pills	Dezhou, Shandong	Offline	*Boschniakia rossica*
ZCY56	Shihu Yeguang pills	Dezhou, Shandong	Offline	*Cistanche tubulosa*
ZCY57	Shihu Yeguang pills	Guangzhou, Guangdong	Offline	*Boschniakia rossica*
ZCY58	Shihu Yeguang pills	Guangzhou, Guangdong	Online	*Cistanche tubulosa*
ZCY63	Yucong Qiangshen capsules	Guangzhou, Guangdong	Online	*Cistanche deserticola, Cistanche tubulosa*
ZCY64	Shihu Yeguang pills	Shenzhen, Guangdong	Offline	*Cynomorium songaricum, Cistanche deserticola*
ZCY65	Shihu Yeguang pills	Shenzhen, Guangdong	Offline	*Cistanche deserticola*
ZCY66	Wenweishu capsules	Beijing	Offline	*Cistanche deserticola*
ZCY69	Shihu Yeguang pills	Wenzhou, Zhejiang	Online	*Cistanche deserticola, Cistanche tubulosa*
ZCY70	Shihu Yeguang pills	Jiaxing, Zhejiang	Online	*Cistanche deserticola, Cistanche tubulosa*
ZCY71	Shihu Yeguang pills	Jiaxing, Zhejiang	Online	*Cistanche tubulosa*
ZCY72	Shihu Yeguang pills	Guangzhou, Guangdong	Offline	*Cistanche tubulosa*
ZCY74	Shihu Yeguang pills	Kunming, Yunnan	Online	*Cynomorium songaricum, Cistanche tubulosa*
ZCY79	Sanbao capsules	Dongguan, Guangdong	Online	*Cynomorium songaricum*
ZCY85	Shihu Yeguang pills	Bozhou, Anhui	Offline	*Cistanche tubulosa*
ZCY92	Shihu Yeguang pills	Chengdu, Sichuan	Offline	*Cistanche tubulosa*
ZCY94	Sanbao capsules	Chengdu, Sichuan	Offline	*Cynomorium songaricum, Cistanche deserticola, Cistanche sinensis*
ZCY95	Shihu Yeguang pills	Chengdu, Sichuan	Offline	*Cistanche tubulosa*
ZCY96	Shihu Yeguang pills	Chengdu, Sichuan	Offline	*Cistanche tubulosa*

Mixed samples: Powders of *Ci. deserticola, Ci. tubulosa, Cy. songaricum, Ci. sinensis, B. rossica*, and *O. coerulescens* were artificially mixed in different combinations (at a ratio of 1:1) before extraction. The details of these mixed samples are shown in the legend of Figure **3**. In addition, the powders of four adulterants were mixed with the genuine *Ci. deserticola* at different weight ratios: 10:1, 50:1, 100:1, 200:1, 500:1, 1000:1, 2000:1, 5000:1, 10000:1, 15000:1, 20000:1, 25000:1, 30000:1, 40000:1, 50000:1 and 60000:1 (Table 3). And the two genuine products are mixed in the same proportions.

**Table 3:** qRT-PCR Cq values with standard deviation for mixtures at different sample weight ratios.

**Detected species**	**Sample composition of mixture**	**Cq values with standard deviation of different sample weight ratios**							
		**10:1**	**50:1**	**100:1**	**200:1**	**500:1**	**1000:1**	**2000:1**	**5000:1**
*Cy. songaricum*	*Ci. deserticola*: *Cy. songaricum*	24.92 ± 0.188	27.1 ± 0.022	27.81 ± 0.147	29.24 ± 0.394	30.47 ± 0.304	31.39 ± 0.561	31.42 ± 0.406	32.46 ± 0.862
*Ci. sinensis*	*Ci. deserticola*: *Ci. sinensis*	23.64 ± 0.078	26.00 ± 0.101	27.07 ± 0.155	28.26 ± 0.109	29.46 ± 0.066	30.47 ± 0.125	31.36 ± 0.101	33.36 ± 0.190
*B. rossica*	*Ci. deserticola*: *B. rossica*	18.27 ± 0.138	21.32 ± 0.821	22.28 ± 0.033	25.78 ± 0.020	26.25 ± 0.031	27.22 ± 0.052	29.33 ± 0.823	30.45 ± 0.262
*O. coerulescens*	*Ci. deserticola*: *O. coerulescens*	19.07 ± 0.022	21.34 ± 0.030	22.53 ± 0.105	23.77 ± 0.111	25.16 ± 0.089	26.36 ± 0.098	27.30 ± 0.164	29.25 ± 0.049
*Ci. tubulosa*	*Ci. deserticola*: *Ci. tubulosa*	24.05 ± 0.145	26.93 ± 0.176	27.97 ± 0.044	29.56 ± 0.161	30.60 ± 0.338	31.18 ± 0.090	32.25 ± 0.122	33.70 ± 0.222
*Ci. deserticola*	*Ci. tubulosa*: *Ci. deserticola*	23.93 ± 0.165	25.06 ± 0.155	26.25 ± 0.093	28.05 ± 0.085	29.07 ± 0.163	30.04 ± 0.194	31.82 ± 0.107	32.90 ± 0.424
		**10000:1**	**15000:1**	**20000:1**	**25000:1**	**30000:1**	**40000:1**	**50000:1**	**60000:1**
*Cy. songaricum*	*Ci. deserticola*: *Cy. songaricum*	34.48 ± 0.255	35.83 ± 0.328	None	None	None	None	None	None
*Ci. sinensis*	*Ci. deserticola*: *Ci. sinensis*	34.33 ± 0.214	35.45 ± 0.125	36.26 ± 0.756	None	None	None	None	None
*B. rossica*	*Ci. deserticola*: *B. rossica*	31.98 ± 0.804	32.79 ± 0.361	33.80 ± 0.550	35.46 ± 0.886	37.08 ± 0.162	None	None	None
*O. coerulescens*	*Ci. deserticola*: *O. coerulescens*	30.01 ± 0.260	31.36 ± 0.284	32.84 ± 0.338	33.52 ± 0.215	36.38 ± 0.459	36.16 ± 0.509	None	None
*Ci. tubulosa*	*Ci. deserticola*: *Ci. tubulosa*	34.90 ± 0.603	35.95 ± 0.470	36.48 ± 0.501	None	None	None	None	None
*Ci. deserticola*	*Ci. tubulosa*: *Ci. deserticola*	35.17 ± 0.183	35.23 ± 0.544	36.25 ± 0.279	None	None	None	None	None

Decoction: Slices of *Ci. deserticola* and *Cy. songaricum* were used to prepare the decoction. The slices (10 g) were boiled in 300 mL of double-distilled water for 30, 60, 90, 120, 150, 180, 210, and 240 min and then used for DNA extraction.

### DNA extraction, polymerase chain reaction (PCR) amplification and sequencing

Specimens, decoction, and mixed samples: The samples (40–50 mg) were ground into fine powders via a Retsch MM400 laboratory mixer mill (Retsch Co., Germany) at a frequency of 30 Hz. The genomic DNA was subsequently extracted with a Plant Universal Genomic DNA Kit (Tiangen Biotech Beijing Co., China) according to the manufacturer's instructions. ITS2 was amplified by the universal primers 2F/3R (Chen et al., 2010).

Extract and Chinese patent medicines: Samples (40–50 mg) were collected into a tube and then ground via a Retsch MM400 laboratory mixer mill (Retsch Co.). Ten samples were collected in parallel per batch. The Chinese patent medicine powder was washed with 700 μL of prewash buffer [100 mM Tris-HCl, pH 8.0; 20 mM ethylenediaminetetraacetic acid (EDTA), pH 8.0; 700 mM NaCl; 2% polyvinylpyrrolidone (PVP)-40; and 0.4% β-mercaptoethanol] several times until the supernatant was clear and colorless, after which the mixture was centrifuged at 7500 × g for 5 min at room temperature. The precipitate was subsequently used to extract the genomic DNA via the Plant Universal Genomic DNA Kit (Tiangen Biotech Beijing Co.) according to the manufacturer's instructions. In the end, DNA from each batch was concentrated into one tube.

Six species-specific primer pairs—SYF1/SYR1, HMRCF/HMRCR, GHRCF/GHRCR, CCRF/CCRR, SCRF/SCRR, and LDF/LDR—were designed via Primer Premier 6.0 software (Premier Co., Canada) to amplify Cistanches Herba and its adulterants (the details are shown in Table 4). PCR was performed in a 50 μL-volume reaction containing 1 μL of KOD FX (Toyobo Co., Japan), 25 μL of 2 × PCR buffer, 10 μL of dNTPs (2 mM), 1.5 μL of each primer (10 μM), and 4 μL (~50 ng) of DNA template; the remaining volume consisted of double-distilled water. The reactions were performed by a thermal cycler (Veriti™ 96-Well Thermal Cycler, Applied Biosystems Co., USA); the thermal programs are listed in Table 4. The PCR products were examined via 3% agarose gel electrophoresis and purified for bidirectional sequencing with an ABI 3730XL sequencer (Applied Biosystems Co.) in accordance with the Sanger sequencing method. Then, the sensitivity of the six primers was tested via the quantitative real-time PCR (qRT-PCR) assay with a CFX96 Real-time System (Bio-rad Lab., USA). The cycle thresholds were automatically calculated by the system via the “PCR Baseline Subtracted Curve Fit” model. qRT-PCR was performed in a 15 μL-volume reaction containing 7.5 μL of SYBR® *Premix Ex Taq*™ (Tli RNaseH Plus) (Takara Bio Co., Japan), 1.0 μL of each primer (10 μM), and 1.0 μL of DNA template with the remaining volume consisting of double-distilled water. The qRT-PCR was performed with three technical replicates based on which standard error was calculated.

**Table 4:** Primers used for PCR amplification and sequencing.

**Primer pair**	**Primer name**	**Direction**	**Primer sequences (5′-3′)**	**Amplicon size**	**Target species**	**Thermal program**
1	SYF1	Forward	CTCAATGTGCTGCTGCTT	123	*Cy. songaricum*	94°C for 2 min;
	SYR1	Reverse	AGACTTACCGCTCACAATG			98°C for 10 s,
2	HMRCF	Forward	CCTTTAGGGTGATACTTAGGT	132	*Ci. deserticola*	50°C for 30 s,
	HMRCR	Reverse	CAGCACGAGAGTTGAGAG			68°C for 15 s, 35 cycles;
3	GHRCF	Forward	TTCTGGGACAATGCTTAGG	134	*Ci. tubulosa*	
	GHRCR	Reverse	CGACACGAGAGTTGAGTT			
4	SCRF	Forward	ATATGGGCGATAGGTAGGT	131	*Ci. sinensis*	
	SCRR	Reverse	GACAGCACGAGAGTTGAG			
5	CCRF	Forward	CGGTCCAAATACGATCCC	72	*B. rossica*	
	CCRR	Reverse	GACAGCACGAGAGTTGAG			
6	LDF	Forward	ATCTTCAACTCTCGTCTGTC	71	*O. coerulescens*	
	LDR	Reverse	CTCGTGCCTATGGGTCTA			

Sequence analysis: The sequences were edited and manually assembled via CodonCode Aligner 5.1.4 (CodonCode Co., USA). ITS sequences from GenBank were annotated via the Hidden Markov model (HMM) to obtain the ITS2 sequences (Keller et al., 2009). The sequences were then aligned by MEGA 5.0 software via the “Muscle” alignment method (Edgar, 2004; Tamura et al., 2011).

## Results

### Development of nucleotide signatures and species-specific primer pairs for cistanches herba and adulterants

**Figure 2:**
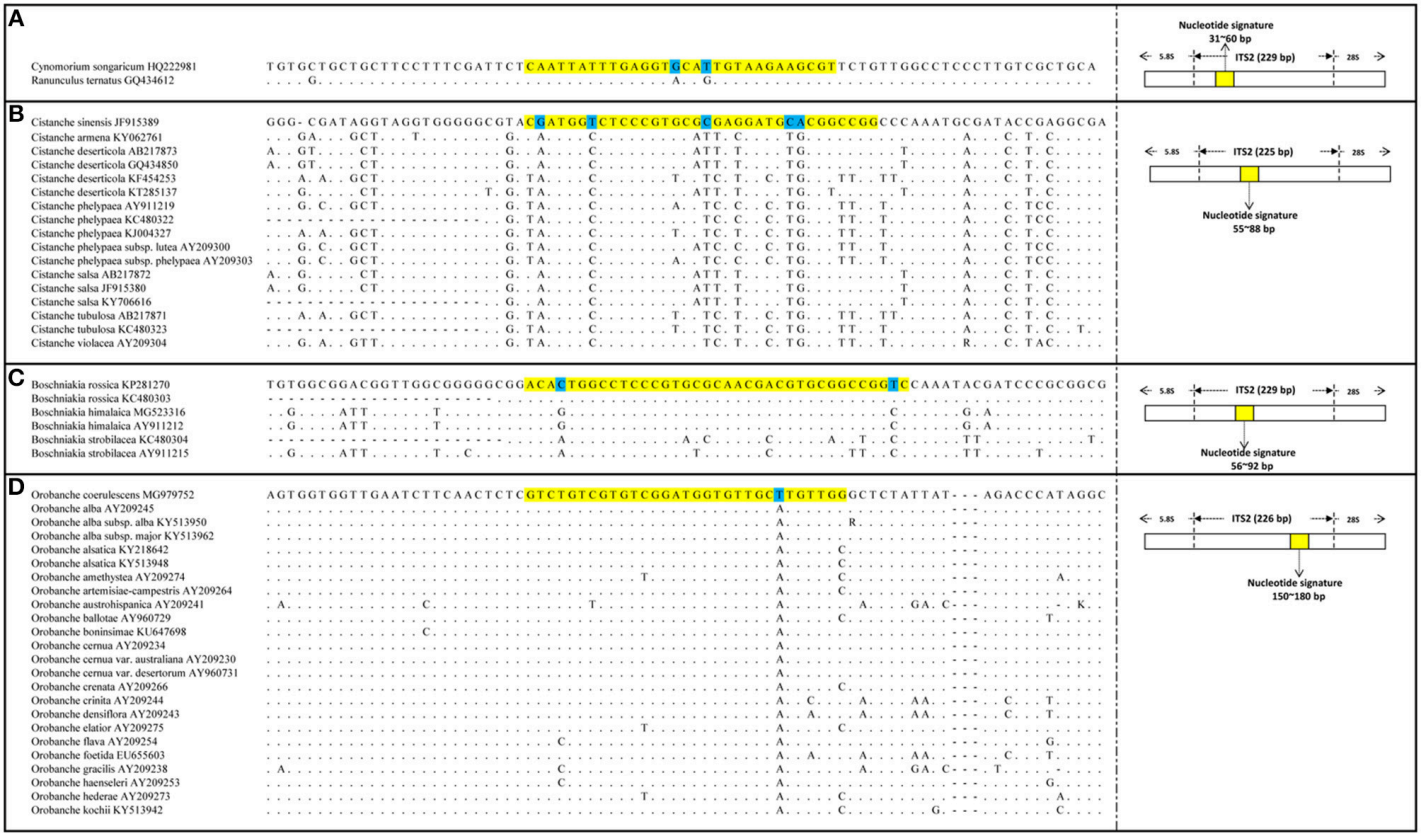
DNA sequence alignment results and SNP sites of the four nucleotide signatures. **(A)** Alignment of *Cynomorium songaricum* nucleotide signature with its region location in ITS2; **(B)** Alignment of *Cistanche sinensis* nucleotide signatures with its region location in ITS2; **(C)** Alignment of *Boschniakia rossica* nucleotide signature with its region location in ITS2; **(D)** Alignment of *Orobanche coerulescens* nucleotide signature with its region location in ITS2. The highlighted regions represent nucleotide signature regions and the marked bases represent the SNP sites of each nucleotide signature, the dots represent identical nucleotides.

The PCR amplification and sequencing success rates of the 251 samples were 100% when the primer pair 2F/3R was used. The aligned length of the *Cy. songaricum* ITS2 sequences was 229 bp (Supplementary Figure S1). Analysis of the sequences from the herbarium species and those of closely related species retrieved from GenBank (Supplementary Table S2) revealed two single nucleotide polymorphism (SNP) sites for *Cy. songaricum*. On the basis of the SNPs, one *Cy. songaricum*-specific 30-bp nucleotide signature (5′-caattatttg aggtgcattg taagaagcgt-3') was developed (Figure 2A). Basic Local Alignment Search Tool (BLAST) results in NCBI demonstrated that this nucleotide signature was unique to *Cy. songaricum* (Table 5). With the similar analysis of the sequences from closely related species, one to two SNPs were discovered from *Ci. sinensis, B. rossica* and *O. coerulescens*. On the basis of the SNPs, the nucleotide signatures for the other three adulterants were also developed similarly, including a 34 bp signature (5′-cgatggtctc ccgtgcgcga ggatgcacgg ccgg-3′) for *Ci. sinensis*, a 37 bp signature (5′-acactggcct cccgtgcgca acgacgtgcg gccggtc-3') for *B. rossica*, and a 31 bp signature (5′-gtctgtcgtg tcggatggtg ttgcttgttg g-3′) for *O. coerulescens* (Figures 2B–D). BLAST analysis in NCBI also revealed that these nucleotide signatures were specific and not present in any other species (Table 5).

**Table 5:** BLAST results of the four conserved nucleotide regions.

**Source species of nucleotide signature**	**Blasted result in NCBI**	**Number of the species**	**Max score**	**Total score**	**Query cover (%)**	**E-value**	**Ident (%)**
*Cynomorium songaricum*	*Cynomorium songaricum*	29	60.0	60.0	100	1e−06	100
	*Bacillus subtilis* strain	3	44.1	44.1	73	0.085	100
	*Ranunculus ternatus*	1	44.1	44.1	100	0.085	93
	*Spirometra erinaceieuropaei*	1	42.1	42.1	83	0.34	96
	*Laccaria bicolor*	1	40.1	40.1	66	1.3	100
*Cistanche sinensis*	*Cistanche sinensis*	4	63.9	63.9	100	5e−08	100
*Boschniakia rossica*	*Boschniakia rossica*	7	69.4	69.4	100	2e−09	100
	*Boschniakia himalaica*	2	60.2	60.2	94	1e−06	97
*Orobanche coerulescens*	*Orobanche coerulescens*	7	58.4	58.4	100	1e−06	100

Cistanches Herba and its adulterants could be amplified simultaneously from mixtures via the 2F/3R universal primer pair. Thus, we designed species-specific primers for nucleotide signature amplification by aligning the ITS2 sequences. Four specific primer pairs—SYF1/SYR1, CCRF/CCRR, SCRF/SCRR, and LDF/LDR—were designed to amplify the nucleotide signatures of *Cy. songaricum, B. rossica, Ci. sinensis*, and *O. coerulescens*, respectively (Table 4). The lengths of the amplicons were 123, 72, 131, and 71 bp, respectively.

In addition, a total of 214 ITS2 sequences from experimental Cistanches Herba materials were analyzed. Two short specific primers—HMRCF/HMRCR and GHRCF/GHRCR for *Ci. deserticola* and *Ci. tubulosa*, respectively—were designed to amplify the short regions of the degraded samples(Table 4). The lengths of the amplicons were 132 and 134 bp, respectively.

### Validation of the nucleotide signature and species-specific primer method based on artificial mixtures and decoction

**Figure 3:**
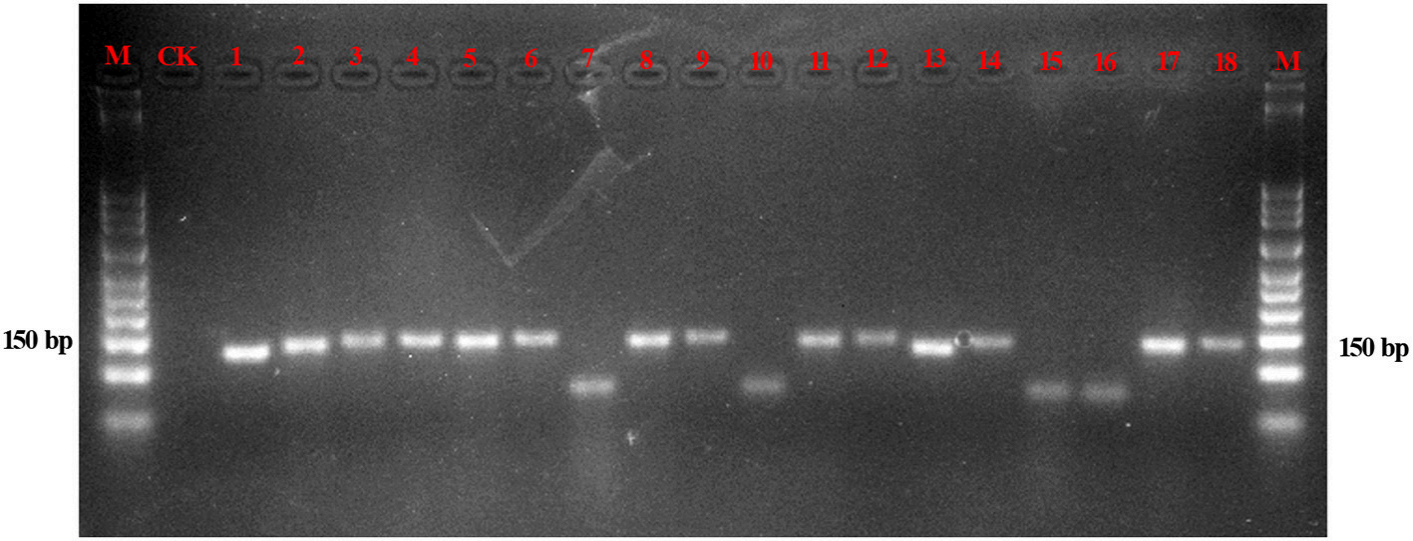
Gel image of the products of PCR amplification of the mixed powders. With the exception of the lanes marked M (marker) and CK (negative control), the other 13 lanes contain the PCR products of the mixtures. Lanes 1–3 are a mixture of *Cy. songaricum, Ci. deserticola* and *Ci. tubulosa* amplified with the primers SYF1/SYR1, HMRCF/HMRCR, and GHRCF/GHRCR, respectively; lanes 4–6 are a mixture of *Ci. sinensis, Ci. deserticola*, and *Ci. tubulosa* amplified with the primers SCRF/SCRR, HMRCF/HMRCR, and GHRCF/GHRCR, respectively; lanes 7–9 are a mixture of *B. rossica, Ci. deserticola*, and *Ci. tubulosa* amplified with the primers CCRF/CCRR, HMRCF/HMRCR, and GHRCF/GHRCR, respectively; lanes 10–12 are a mixture of *O. coerulescens, Ci. deserticola*, and *Ci. tubulosa* amplified with the primers LDF/LDR, HMRCF/HMRCR, and GHCRF/GHRCR, respectively; and lanes 13–18 are a mixture of *Cy. songaricum, Ci. sinensis, B. rossica, O. coerulescens, Ci. deserticola*, and *Ci. tubulosa* with the primers SYF1/SYR1, SCRF/SCRR, CCRF/CCRR, LDF/LDR, HMRCF/HMRCR, and GHCRF/GHRCR, respectively.

The amplification efficiencies of the new primer pairs were validated from the mixture. PCR products were obtained via each primer pair for each targeted species, as shown in Figure 3. For instance, the fifth mixture sample is a mixture of Cistanches Herba and its four adulterants, and each species could be amplified with the primers SYF1/SYR1, SCRF/SCRR, CCRF/CCRR, LDF/LDR, HMRCF/HMRCR, and GHRCF/GHRCR. Moreover, the amplification regions were sequenced, and the nucleotide signatures were observed within the target sequences. Furthermore, to measure the sensitivity, mixtures of two of the six samples were created at different weight ratios and the sample with lower proportion was amplified by specific primer pairs via qRT-PCR. As shown in Table 3, cq values of *B. rossica* or *O. coerulescens* could be obtained even the proportion of samples was 30000:1 or 40000:1, and the amplification results of *Ci. sinensis, Ci. tubulosa*, and *Ci deserticola* could be detected when the ratio was 20000:1. But no detectable results could be obtained for *Cy. songaricum* when it constituted the proportion of 20000:1.

**Figure 4:**
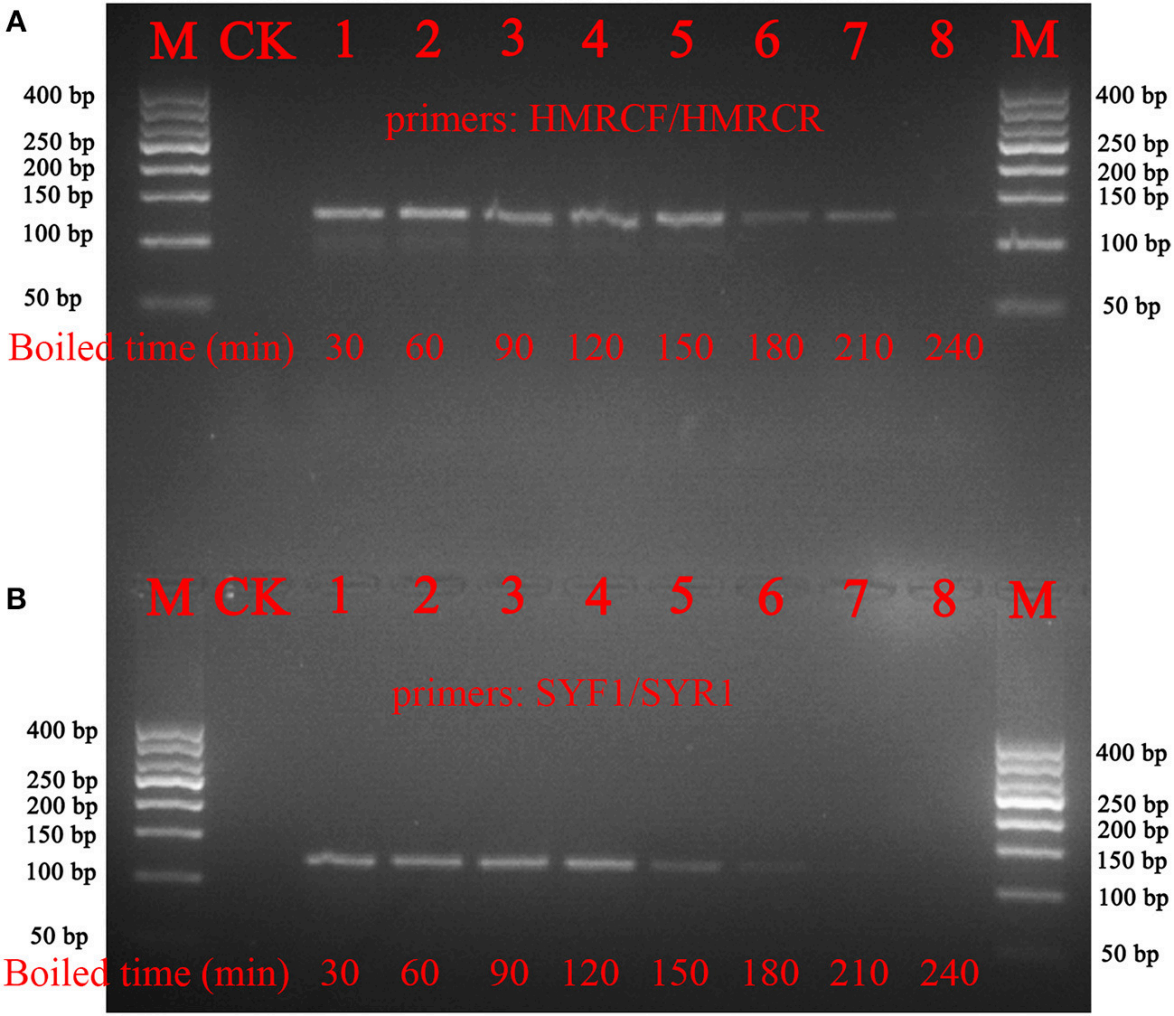
Gel images of the PCR amplifications of the boiled samples of *Ci. deserticola* and *Cy. Songaricum*. **(A)** *Ci. deserticola*; **(B)** *Cy. Songaricum*. With the exception of the lanes marked M (marker) and CK (negative control), the remaining 8 lanes contain the PCR products from the samples boiled for 30, 60, 90, 120, 150, 180, 210, and 240 min, respectively.

To verify whether the nucleotide signature method functions with processed materials, decoctions of *Ci. deserticola* and *Cy. songaricum* were prepared. The results showed that the short barcode from *Ci. deserticola* could be amplified, even after the samples were boiled for 210 min. In addition, the nucleotide signature of *Cy. songaricum* could be amplified after the samples were boiled for 150 min, while no PCR products were detected after the samples were boiled for 210 or 240 min (Figure 4). The sequencing results demonstrated that the short nucleotide signature was successfully obtained from the decoction.

### Market survey of adulteration via nucleotide signature and specific primers

The above method was applied for the detection of Cistanches Herba products on the market. Thirty five batches of Cistanches Herba slices, powders and extracts were amplified and sequenced by using six designed specific primers (the agarose gel electrophoresis results are shown in Supplementary Figure S2 and the sequences are listed in Supplementary Data Sheet 1). Analysis of the sequences via their nucleotide signatures revealed that five batches of slices were authentic. One slice batch and one powder batch were substituted with *Cy. songaricum*, and one extract was substituted with *Ci. sinensis*. The other six batches of powders were mixtures: five were adulterated with *Cy. songaricum* and *Ci. sinensis*, and one was adulterated with *Cy. songaricum* (Table 1). In addition, one slice batch was substituted with *Salvia miltiorrhiza*.

The Cistanches Herba in Chinese patent medicines is subjected to various processes that make authentication difficult. The ability of the six primer pairs to amplify the species-specific nucleotide signature regions from the Chinese patent medicines was tested (the agarose gel electrophoresis results are shown in Supplementary Figure S2). Most Chinese patent medicines contain components of different types of species. For example, Shihu Yeguang pills (ZCY34) contain 25 ingredients, including Cistanches Herba (*Rou Cong Rong*), Dendrobii Caulis (*Shi Hu*), and Ginseng Radix et Rhizoma (*Ren Shen*). From these ingredients, Cistanches Herba could be amplified specifically via the primer pair GHRCF/GHRCR. Direct sequencing of the PCR products revealed very clean traces. However, a visible band could not be obtained with the primer pair HMRCF/HMRCR.

Another example is Kangguzhi Zengsheng pills (ZCY44). There are eight ingredients in addition to Cistanches Herba in this Chinese patent medicine, but there were no visible bands obtained by either GHRCF/GHRCR or HMRCF/HMRCR, which meant that no Cistanches Herba was present. However, the adulterant region of *Cy. songaricum* was successfully amplified by the specific primer pair SYF1/SYR1. The sequencing results demonstrated the short nucleotide signature of *Cy. songaricum* was successfully obtained.

By seeking the nucleotide signatures developed in this study, we found that 15 of 31 Chinese patent medicines labeled as containing Cistanches Herba instead contained adulterants, including eight counterfeit ingredients and seven adulterants (Table 2). For example, six batches were replaced with *Cy. songaricum*, including three batches of Shihu Yeguang pills, one batch of Kangguzhi Zengsheng pills, one batch of Sanbao capsules, and one batch of Wenweishu particles.

Moreover, different batches from the same manufacturer produced somewhat different results. For example, among three batches from one manufacturer (ZCY16, ZCY69, and ZCY71), one batch comprised a mixture of *Ci. deserticola* and *Cy. songaricum*, one batch comprised a mixture of *Ci. deserticola* and *Ci. tubulosa*, and one batch contained only *Ci. tubulosa*. Two batches from another manufacturer (ZCY44 and ZCY70) also differed: one batch contained *Cy. songaricum*, whereas the other batch contained *Ci. deserticola* and *Ci. tubulosa*.

Cistanches Herba was detected in 23 of the 31 Chinese patent medicines tested (Table 2), and only 16 samples were authentic, e.g., without adulterants or counterfeit ingredients. *Ci. tubulosa* was detected in 16 batches of Chinese patent medicines, and *Ci. deserticola* was detected in 10 batches. *O. coerulescens* was not detected in any of the products (Table 2).

## Discussion

### Necessity of developing a new method for monitoring commercially available medicinal products containing cistanches herba

Cistanches Herba is a tonic that is widely used in restorative Chinese patent medicines and other medicinal products. However, the quality control of Chinese patent medicines presents great challenge due to the diversity and complexity of the ingredients. Due to the lack of regulatory oversight, there is considerable opportunity for product adulteration or counterfeiting. In addition, all products should be processed in accordance with the Pharmacopoeia or other standards; adulterating or counterfeiting is not permitted during processing. Thus, varieties of quality control methods have been established, such as multi-heart-cutting two-dimensional liquid chromatography (Yao et al., 2015), near-infrared reflectance spectroscopy (Zhang and Su, 2014; Zhang et al., 2015) and liquid chromatography-mass spectrometry (Wang et al., 2016b). However, the analytical chemistry methods currently in the Chinese Pharmacopoeia Commission (2015) cannot be used to authenticate all of the ingredients in Chinese patent medicines or to detect the presence of adulterant ingredients. Moreover, studies have shown that targeted metabolites in plants are altered during product processing, resulting in considerable variability in test results or complete failure of test methods (Ananingsih et al., 2013). Thus, molecular tools such as species-specific nucleotide signatures are poised to reinforce quality control systems against the risk of fraudulent product substitution and adulteration and inclusion of unlabeled ingredients.

Although ITS/ITS2 is considered a high-efficiency tool for the identification of herbal medicines, these sequences cannot be amplified from highly processed samples (Newmaster et al., 2013; de Boer et al., 2015). Wang et al. reported that ITS2 could not be amplified from Angelicae Sinensis Radix extract or decoctions boiled for more than 120 min (Wang et al., 2016a). According to traditional technologies and the Chinese Pharmacopoeia Commission (2015), Cistanches Herba is always highly processed to increase its medicinal efficacy; these processes, include oven drying, salting and steaming with wine (Zou et al., 2017), which lead to DNA degradation. In addition, various excipients are added during processing, such as honey, starch, and dextrin. If these excipients are not removed completely, the purity of DNA will be affected. For example, the following manufacturing process is used to generate Cistanches Herba-containing Sanbao capsules: “Boil the medicinal slices for 1.5 h twice, combine the decoctions and filter the mixture. Concentrate the filtrate to a relative density of 1.20~1.25 (at 80°C). Add other ground powders and combine them to obtain a homogeneous mixture. Next, dry the mixture at 60°C, and then grind it into a fine powder.” However, after the production process described above, there could be difficulties during the DNA extraction of Chinese patent medicines, and long fragments might not be amplified from the degraded DNA, which would prevent the identification of adulterants. Thus, to ensure the quality and purity of DNA, we added additional steps before the genomic DNA extraction, including washing with prewash buffer and eluting ten parallel tubes into one tube for each batch.

Although all the Chinese patent medicines used in the present study contained 6–25 ingredients, the primer pairs developed could specifically amplify the sequences of the adulterants in these Chinese patent medicines. Direct sequencing of the PCR products showed clean trace files. Thus, this nucleotide signature method is capable of identifying both authentic species ingredients and adulterants and should broaden the application of DNA-based molecular diagnostic tools for market supervision.

### Nucleotide signatures for the effective identification of cistanches herba products

Molecular tools that utilize PCR technology are very promising for medicinal product authentication within quality control systems. The successful application of the primers for identifying DNA-degraded adulterants from Cistanches Herba suggests that a PCR-based detection method could be used widely. In the Chinese herbal medicine market, the price of authentic Cistanches Herba species ingredients is more than five times higher than the prices of its adulterants. Our results showed that *Cy. songaricum* is the most common adulterant of Cistanches Herba on the market, followed by *Ci. sinensis*. *Cy. songaricum* was added because these medicines share similar morphological characteristics. In addition, the chemical composition of *Ci. sinensis* is similar to that of Cistanches Herba. As quality control markers for Cistanches Herba extracts, echinacoside and acteoside can be inexpensively extracted from *Ci. sinensis*. Thus, some pharmaceutical factories use *Ci. sinensis* as a substitute in the production of Cistanches Herba extracts. Taken together, the results of this study indicate that there is considerable fraud in the market for medicinal products.

Adulteration in Chinese patent medicine is similar to that found in other countries. Similar levels of adulteration have been recorded in North America (Newmaster et al., 2013), Europe (Raclariu et al., 2017), and Asia (Cheng et al., 2014; Shanmughanandhan et al., 2016; Gao et al., 2017). In this study, the adulterated rate of Chinese patent medicines was approximately 48.4%, with only 16 of the 31 samples being authentic Cistanches Herba. In addition, we speculated that the different results produced in products from the same manufacturer could be attributed to differences in the qualities of the different batches of Chinese medicine materials. Therefore, to control the quality of Chinese patent medicines, the raw materials should be authenticated before being processed into products.

Adulteration of Cistanches Herba has traditionally been associated with issues of supply and demand of raw materials. *Ci. deserticola* and *Ci. tubulosa* are the two original plants currently used to formulate Cistanches Herba. However, *Ci. deserticola* is the only original species in traditional authentic Cistanches Herba listed in the Chinese Pharmacopoeia Commission (2000), in which *Ci. tubulosa* is identified as an adulterant. Owing to the shortage of *Ci. deserticola* resources, *Ci. tubulosa* has been listed as a supplement in the Chinese Pharmacopoeia since 2005 (Jiang and Tu, 2009). Until recently, the prices of these herbs have markedly differed; *Ci. tubulosa* has been much less expensive than *Ci. deserticola* because there is a much larger supply of the former. Here, our results showed that *Ci. tubulosa* is more widely used in commercially available Cistanches Herba products.

In conclusion, the nucleotide signatures and PCR-based methods developed in this study may serve as useful tools for the medicinal product industry to authenticate ingredients and detect adulterants in Cistanches Herba products. In accordance of the sensitivity result, even if the proportion of adulterant was one in ten thousand, it can be detected via qRT-PCR. It means that once a nucleotide signature is detected in Cistanches Herba-containing functional products, it could be identified as an adulterant or counterfeit ingredient. A novel solution for detecting counterfeit ingredients or adulterated Cistanches Herba was provided that was not previously available via chemical detection methods in the Chinese Pharmacopoeia. In addition, this method could be used to validate increasing types of medicine and to broaden the applications of DNA-based molecular diagnostic tools for market supervision.

## Author contributions

JH conceived the study and participated in its design. XW, RX, and JC contributed samples and performed the experiments. XW analyzed the data. XW, JH, ZZ, S-GN, JS, and SC drafted the manuscript. All authors have read and approved the final manuscript.

### Conflict of interest statement

The authors declare that the research was conducted in the absence of any commercial or financial relationships that could be construed as a potential conflict of interest.

## Supplementary material

Supplementary Figure S1 The alignment result of ITS2 sequence from six species.

Supplementary Figure S2 Agarose gel electrophoresis of all samples.

Supplementary Table S1 Sampling information of Cistanches Herba and its adulterants.

Supplementary Table S2 Sequence information of related species downloaded from GenBank.

Supplementary Table S3 The declared compositions of different Chinese patent medicine samples.

Supplementary Data Sheet S1 All the sequences obtained in this study.
