# Supplementary material for: Detection of Cistanches Herba (Rou Cong Rong) Medicinal Products Using Species-Specific Nucleotide Signatures
Source: Front Plant Sci. 2018 Nov 13;9:1643. doi: 10.3389/fpls.2018.01643 (PMC6242781; doi:10.3389/fpls.2018.01643)
Supplement: Supplementary Figure S1 — The alignment result of ITS2 sequence from six species. [file Image_1.pdf]

Cistanche deserticola YC0089MT15	C G C A T C G C G T C G C T C C C C T C C C G T C C C T T T A G G G T G A T A C T T A G G T G G G G G C G G A C A A T G G C C T C C C G T G C A T T A T T G A T G T G C G G C C G G C C T A A A T G A G A T C C T G C G G C G A C T C A C G T C	[120
Cistanche deserticola YC0089MT14	. . . .	[120
Cistanche deserticola YC0089MT13	. . . .	[120
Cistanche deserticola YC0089MT12	. . . .	[120
Cistanche deserticola YC0089MT11	. . . .	[120
Cistanche deserticola YC0089MT10	. . . .	[120
Cistanche deserticola YC0089MT09	. . . .	[120
Cistanche deserticola YC0089MT08	. . . .	[120
Cistanche deserticola YC0089MT05	. . . .	[120
Cistanche deserticola QT8-4	. . . .	[120
Cistanche deserticola QT6-7	. . . .	[120
Cistanche deserticola KT285139	. . . . C . . . . T . . . T . . . . T . . . . A	[120
Cistanche deserticola KT285138	. . . . C . . . . T . . . T . . . . T . . . . A	[120
Cistanche deserticola KT285137	. . . . C . . . . T . . . T . . . . T . . . . A	[120
Cistanche deserticola HT4-5	. . . .	[120
Cistanche deserticola CL1-2	. . . .	[120

Cistanche deserticola YC0089MT15	A C G A C C A G T G G T G G T T G A A C T C T C A A C T C T C G T G C T G T T G T G A C G T A T G G C G T C G A A T G G T A G A C A T T A T C G C A T A C C C A A T G G T G T G A T A T A T T C A C G C T T T T G A C C G	[229]
Cistanche deserticola YC0089MT14	. . . .	[229]
Cistanche deserticola YC0089MT13	. . . .	[229]
Cistanche deserticola YC0089MT12	. . . .	[229]
Cistanche deserticola YC0089MT11	. . . .	[229]
Cistanche deserticola YC0089MT10	. . . .	[229]
Cistanche deserticola YC0089MT09	. . . .	[229]
Cistanche deserticola YC0089MT08	. . . .	[229]
Cistanche deserticola YC0089MT05	. . . .	[229]
Cistanche deserticola QT8-4	. . . . G	[229]
Cistanche deserticola QT6-7	. . . . G	[229]
Cistanche deserticola KT285139	. . . . T . . . . C . . . . G	[229]
Cistanche deserticola KT285138	. . . . T . . . . C . . . . G	[229]
Cistanche deserticola KT285137	. . . . T . . . . C . . . . G	[229]
Cistanche deserticola HT4-5	. . . .	[229]
Cistanche deserticola CL1-2	. . . . G	[229]

Cistanche tubulosa YC0050MT02	C G C A T C G T G T T G C C C C C T C C T C T G T C C T T C T G G G A C A A T G C T T A G G T G G G G G C G G A T A A T G G C C T C C C G T T C G T C A T G A C G T G C G G T T G G T T C A A A T G A G A T C C T G C G G C G A T G C A C G T C	[120
Cistanche tubulosa YC0050MT03	. . . .	[120
Cistanche tubulosa YC0050MT04	. . . .	[120
Cistanche tubulosa YC0050MT05	. . . .	[120
Cistanche tubulosa YC0050MT06	. . . .	[120
Cistanche tubulosa YC0050MT07	. . . .	[120
Cistanche tubulosa YC0050MT08	. . . .	[120
Cistanche tubulosa YC0050MT09	. . . .	[120
Cistanche tubulosa YC0050MT10	. . . .	[120
Cistanche tubulosa YC0050MT11	. . . .	[120
Cistanche tubulosa YC0050MT12	. . . .	[120
Cistanche tubulosa YC0050MT13	. . . .	[120
Cistanche tubulosa AB217871	. . . .	[120
Cistanche tubulosa JF915386	. . . .	[120
Cistanche tubulosa JF915385	. . . .	[120
Cistanche tubulosa JF915384	. . . .	[120
Cistanche tubulosa JF915383	. . . .	[120
Cistanche tubulosa JF915379	. . . .	[120
Cistanche tubulosa GQ434563	. . . .	[120
Cistanche tubulosa GQ434562	. . . .	[120

Cistanche tubulosa YC0050MT02	G T G A C C A G T G G T G G T T G A A C T C T C A A C T C A A C T C T C G T G T C G T T G T G A C G T T T G G C G T T G T G C G G T G G G A T T A T T G C A T A C C C A A T C G T G C G A T C T A T T T C G C G C T T T C G A C G	[233]
Cistanche tubulosa YC0050MT03	. . . . T	[233]
Cistanche tubulosa YC0050MT04	. . . . T	[233]
Cistanche tubulosa YC0050MT05	. . . . T	[233]
Cistanche tubulosa YC0050MT06	. . . .	[233]
Cistanche tubulosa YC0050MT07	. . . . T	[233]
Cistanche tubulosa YC0050MT08	. . . . T	[233]
Cistanche tubulosa YC0050MT09	. . . . T	[233]
Cistanche tubulosa YC0050MT10	. . . . T	[233]
Cistanche tubulosa YC0050MT11	. . . . T	[233]
Cistanche tubulosa YC0050MT12	. . . . T	[233]
Cistanche tubulosa YC0050MT13	. . . . T	[233]
Cistanche tubulosa AB217871	. . . . T . . . . -	[233]
Cistanche tubulosa JF915386	. . . . T	[233]
Cistanche tubulosa JF915385	. . . . T	[233]
Cistanche tubulosa JF915384	. . . . T	[233]
Cistanche tubulosa JF915383	. . . . T	[233]
Cistanche tubulosa JF915379	. . . . T	[233]
Cistanche tubulosa GQ434563	. . . . T	[233]
Cistanche tubulosa GQ434562	. . . . T	[233]

```
Cynomorium songaricum YC16           CTCAATGTGCTGCTGCTTCCTTTCGATTCTCAATTATTTGAGGTGCATTGTAAGAAGCGTTCTGTTGGCCTCCCTTGTCGCTGCAAAGGTTGGCTCAAAAGCTTCATTGTGAGCGGTAAG [120]
Cynomorium songaricum YC14           ........................................................................................................................ [120]
Cynomorium songaricum YC12           ........................................................................................................................ [120]
Cynomorium songaricum YC10           ........................................................................................................................ [120]
Cynomorium songaricum YC08           ........................................................................................................................ [120]
Cynomorium songaricum YC07           ........................................................................................................................ [120]
Cynomorium songaricum YC02           ........................................................................................................................ [120]
Cynomorium songaricum YC0114MT14     ........................................................................................................................ [120]
Cynomorium songaricum YC0114MT12     ........................................................................................................................ [120]
Cynomorium songaricum YC0114MT11     ........................................................................................................................ [120]
Cynomorium songaricum YC0114MT10     ........................................................................................................................ [120]
Cynomorium songaricum YC0114MT09     ........................................................................................................................ [120]
Cynomorium songaricum YC0114MT08     ........................................................................................................................ [120]
Cynomorium songaricum YC0114MT06     ........................................................................................................................ [120]
Cynomorium songaricum YC0114MT05     ........................................................................................................................ [120]
Cynomorium songaricum YC0114MT04     ........................................................................................................................ [120]
Cynomorium songaricum YC0114MT03     ........................................................................................................................ [120]
Cynomorium songaricum YC0114MT02     ........................................................................................................................ [120]
Cynomorium songaricum YC0114MT01     ........................................................................................................................ [120]
Cynomorium songaricum XRSY3          ........................................................................................................................ [120]
Cynomorium songaricum XRSY2          ........................................................................................................................ [120]
Cynomorium songaricum XRSY1          ........................................................................................................................ [120]
Cynomorium songaricum MF096217       ........................................................................................................................ [120]
Cynomorium songaricum MF096216       ........................................................................................................................ [120]
Cynomorium songaricum MF096215       ........................................................................................................................ [120]
Cynomorium songaricum MF096214       ........................................................................................................................ [120]
Cynomorium songaricum KX813705       ........................................................................................................................ [120]
Cynomorium songaricum KX813704       ........................................................................................................................ [120]
Cynomorium songaricum KX813703       ........................................................................................................................ [120]
Cynomorium songaricum KX813702       ........................................................................................................................ [120]
Cynomorium songaricum FDC034         ........................................................................................................................ [120]

Cynomorium songaricum YC16           TCTGCACATTCACTGGTGGGTAGTAAGGCTTGTCCCTTATTGTGGTGTTTGTGTCTGGTCCCTCACTTTTCTCTAGGCCCTGTAAGGTCTTCAACTGGCCCCTTCATTG [229]
Cynomorium songaricum YC14           .......................................................................C......................T.............. [229]
Cynomorium songaricum YC12           .......................................................................C..................................... [229]
Cynomorium songaricum YC10           .......................................................................C......................T.............. [229]
Cynomorium songaricum YC08           .......................................................................C......................T.............. [229]
Cynomorium songaricum YC07           .......................................................................C......................T.............. [229]
Cynomorium songaricum YC02           .......................................................................C......................T.............. [229]
Cynomorium songaricum YC0114MT14     .......................................................................C......................T.............. [229]
Cynomorium songaricum YC0114MT12     .......................................................................C......................T.............. [229]
Cynomorium songaricum YC0114MT11     .......................................................................C......................T.............. [229]
Cynomorium songaricum YC0114MT10     .......................................................................C......................T.............. [229]
Cynomorium songaricum YC0114MT09     .......................................................................C......................T.............. [229]
Cynomorium songaricum YC0114MT08     .......................................................................C......................T.............. [229]
Cynomorium songaricum YC0114MT06     .......................................................................C......................T.............. [229]
Cynomorium songaricum YC0114MT05     .......................................................................C......................T.............. [229]
Cynomorium songaricum YC0114MT04     .......................................................................C......................T.............. [229]
Cynomorium songaricum YC0114MT03     ............................................................................................................. [229]
Cynomorium songaricum YC0114MT02     .......................................................................C......................T.............. [229]
Cynomorium songaricum YC0114MT01     .......................................................................C......................T.............. [229]
Cynomorium songaricum XRSY3          .......................................................................C..................................... [229]
Cynomorium songaricum XRSY2          .......................................................................C..................................... [229]
Cynomorium songaricum XRSY1          .......................................................................C......................T.............. [229]
Cynomorium songaricum MF096217       .......................................................................C......................T.............. [229]
Cynomorium songaricum MF096216       .......................................................................C......................T.............. [229]
Cynomorium songaricum MF096215       ............................................................................................................. [229]
Cynomorium songaricum MF096214       ............................................................................................................. [229]
Cynomorium songaricum KX813705       .......................................................................C......................T.............. [229]
Cynomorium songaricum KX813704       .......................................................................C......................T.............. [229]
Cynomorium songaricum KX813703       .......................................................................C......................T.............. [229]
Cynomorium songaricum KX813702       .......................................................................C......................T.............. [229]
Cynomorium songaricum FDC034         .......................................................................C..................................... [229]
```

```
Boschniakia rossica KP281270         CGCATCGCGTCGCTCCCCCTCCCGTCCCAATGTGGCGGACGGTTGGCGGGGGCGGACACTGGCCTCCCGTGCGCAACGACGTGCGGCCGGTCCAAATACGATCCCGCGGCGACTCACGTC [120]
Boschniakia rossica AY911214         ........................................................................................................................ [120]
Boschniakia rossica MG218819         ........................................................................................................................ [120]
Boschniakia rossica MG219185         ........................................................................................................................ [120]
Boschniakia rossica JF915392         ........................................................................................................................ [120]
Boschniakia rossica JF915381         ........................................................................................................................ [120]
Boschniakia rossica PS2004MT01       ........................................................................................................................ [120]
Boschniakia rossica PS2004MT02       ........................................................................................................................ [120]
Boschniakia rossica KC480303         ............................------------------------.................................................................... [120]

Boschniakia rossica KP281270         ACGACCAGTGGTGGTTGAACACTCAACTCTCGTGCTGTCGTGCCGGATTGTTTCGTATGTCGGGCATTGTCGCATACCCAATGGTGCGAACCATTCGCGCTTCCGACCG [229]
Boschniakia rossica AY911214         ............................................................................--------------------------------- [229]
Boschniakia rossica MG218819         ............................................................................................................. [229]
Boschniakia rossica MG219185         ............................................................................................................. [229]
Boschniakia rossica JF915392         ............................................................................................................. [229]
Boschniakia rossica JF915381         ............................................................................................................. [229]
Boschniakia rossica PS2004MT01       ............................................................................--------------------------------- [229]
Boschniakia rossica PS2004MT02       ............................................................................................................. [229]
Boschniakia rossica KC480303         ..............................................---............................................................ [229]
```

```
Cistanche sinensis AB217874          CGCATCGCGTCGCTCCCCTCCCGTCCATATGGGCGATAGGTAGGTGGGGGCGTACGATGGTCTCCCGTGCGCGAGGATGCACGGCCGGCCCAAATGCGATACCGAGGCGACTCACGTCAC [120]
Cistanche sinensis JF915388          ........................................................................................................................ [120]
Cistanche sinensis JF915389          ........................................................................................................................ [120]
Cistanche sinensis JF915390          ........................................................................................................................ [120]
Cistanche sinensis XRS1              ........................................................................................................................ [120]
Cistanche sinensis XRX2              ........................................................................................................................ [120]
Cistanche sinensis PS2005MT04        ........................................................................................................................ [120]
Cistanche sinensis XRS2              ........................................................................................................................ [120]
Cistanche sinensis PS2005MT01        ........................................................................................................................ [120]
Cistanche sinensis PS2005MT03        ........................................................................................................................ [120]

Cistanche sinensis AB217874          GGCCAGTGGTGGTTGAACACTCAACTCTCGTGCTGTCGTGCACGGCGTCGTACATCGGGTATTTTTGAAAGACCCCACGGCGCGAGCTACTCGCGCTTCAGACC- [225]
Cistanche sinensis JF915388          ........................................................................................................G [225]
Cistanche sinensis JF915389          ........................................................................................................G [225]
Cistanche sinensis JF915390          ........................................................................................................G [225]
Cistanche sinensis XRS1              ........................................................................................................- [225]
Cistanche sinensis XRX2              ........................................................................................................G [225]
Cistanche sinensis PS2005MT04        ........................................................................................................G [225]
Cistanche sinensis XRS2              ........................................................................................................- [225]
Cistanche sinensis PS2005MT01        ........................................................................................................G [225]
Cistanche sinensis PS2005MT03        ........................................................................................................G [225]
```

Orobanche coerulescens AY209235	CACATCGCGTCGCCCTCCCTCCCGTCCCTACGGGGCGTGTTTTGGTGGGGCGGATAATGGCCTCCCGTGCACAACAATGTGCGGCTGGTCYAAATGCGAACCCGCGGCGACTCACGTCAC	[120
Orobanche coerulescens XRL2	....	[120
Orobanche coerulescens KY218643	....T.........C.............................	[120
Orobanche coerulescens KY513935	....C.............................	[120
Orobanche coerulescens JF915391	....C.............................	[120
Orobanche coerulescens PS2006MT01	....C.............................	[120
Orobanche coerulescens JF915382	....T.............................	[120
Orobanche coerulescens XRLD3	....T.............................	[120
Orobanche coerulescens MG979752	............................G...T......A..T.............................	[120
Orobanche coerulescens AY881142	................................T...T.............................	[120
Orobanche coerulescens XRLD5	................................T...T.............................	[120

Orobanche coerulescens AY209235	GACCAGTGGTGGTTGAATCTTCAACTCTCGTCTGTCGTGTCGGATGGTGTTGCTTGTTGGGCTCTATTATAGACCCATAGGCACGAGCTGCTTGTGCTTTCGACTG	[226]
Orobanche coerulescens XRL2	....	[226]
Orobanche coerulescens KY218643	....	[226]
Orobanche coerulescens KY513935	....	[226]
Orobanche coerulescens JF915391	....	[226]
Orobanche coerulescens PS2006MT01	....	[226]
Orobanche coerulescens JF915382	....C.	[226]
Orobanche coerulescens XRLD3	....C.	[226]
Orobanche coerulescens MG979752	....	[226]
Orobanche coerulescens AY881142	....	[226]
Orobanche coerulescens XRLD5	....	[226]
