# Supplementary material for: Detection of Cistanches Herba (Rou Cong Rong) Medicinal Products Using Species-Specific Nucleotide Signatures
Source: Front Plant Sci. 2018 Nov 13;9:1643. doi: 10.3389/fpls.2018.01643 (PMC6242781; doi:10.3389/fpls.2018.01643)
Supplement: Supplementary Figure S2 — Agarose gel electrophoresis of all samples. [file Image_2.pdf]

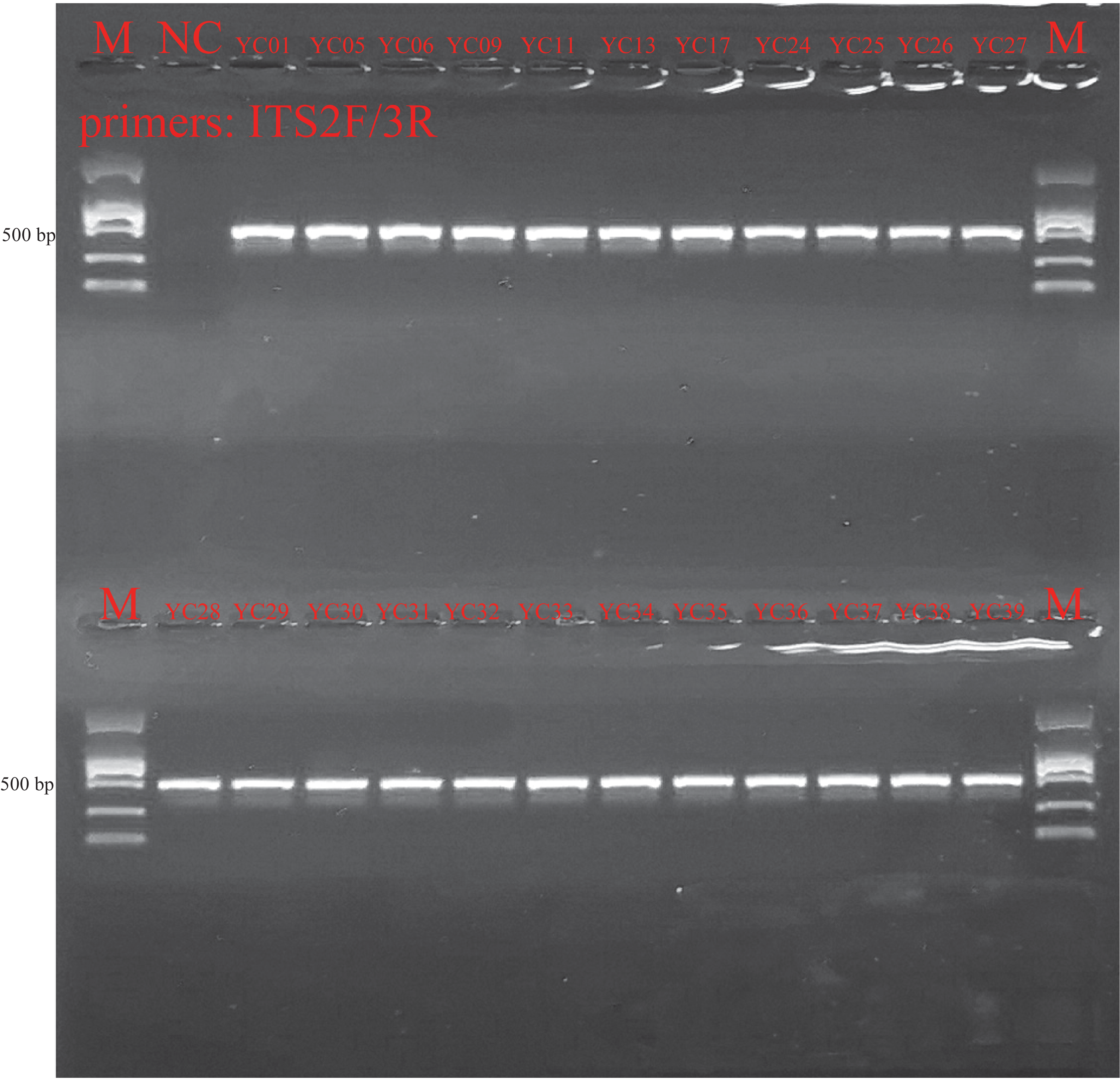
M NC YC01 YC05 YC06 YC09 YC11 YC13 YC17 YC24 YC25 YC26 YC27 M
primers: ITS2F/3R
500 bp
M YC28 YC29 YC30 YC31 YC32 YC33 YC34 YC35 YC36 YC37 YC38 YC39 M
500 bp

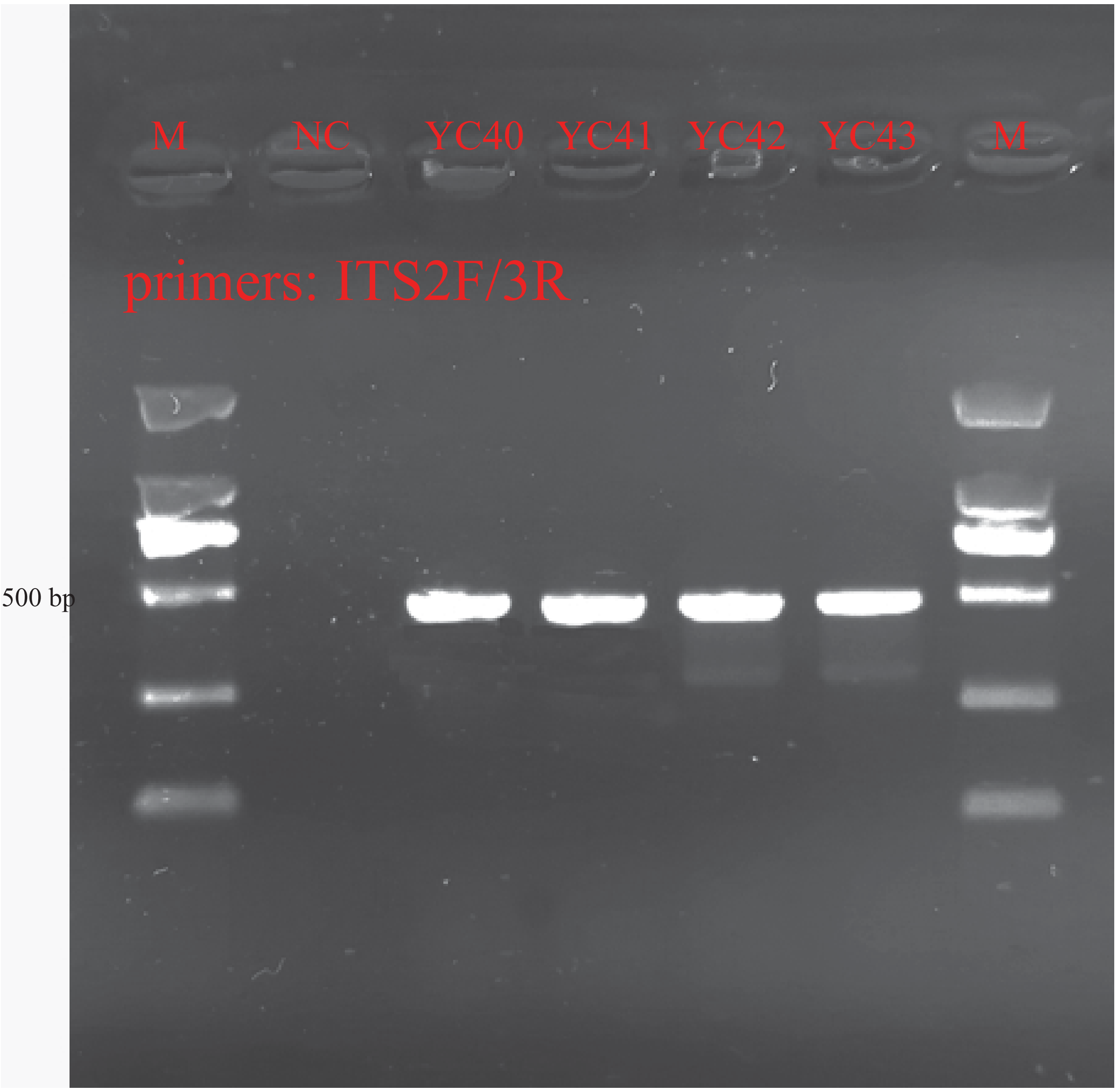
M
NC
YC40
YC41
YC42
YC43
M
primers: ITS2F/3R
500 bp

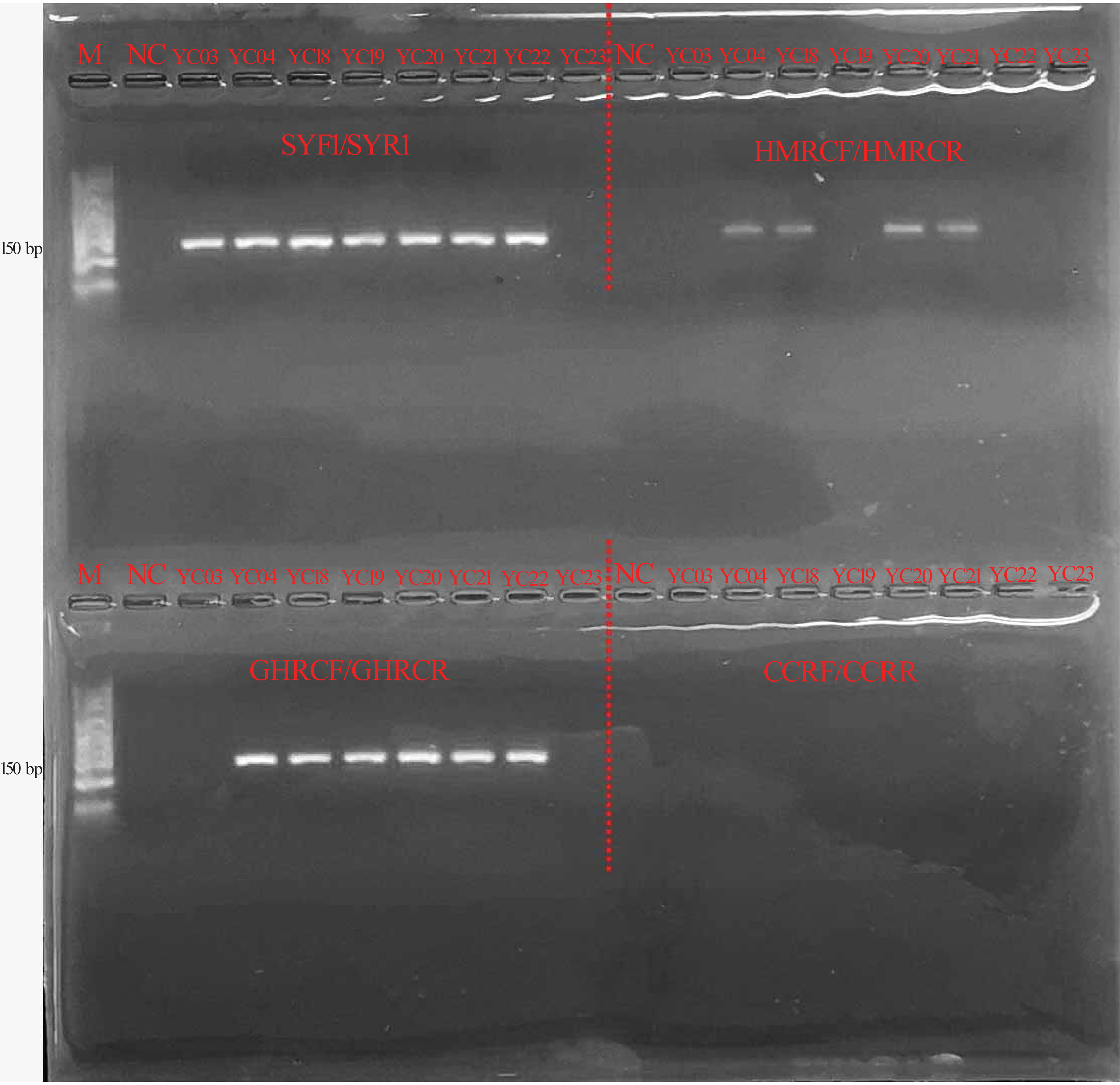
M NC YC03 YC04 YC18 YC19 YC20 YC21 YC22 YC23 NC YC03 YC04 YC18 YC19 YC20 YC21 YC22 YC23
SYF1/SYR1
HMRCF/HMRCR
150 bp
M NC YC03 YC04 YC18 YC19 YC20 YC21 YC22 YC23 NC YC03 YC04 YC18 YC19 YC20 YC21 YC22 YC23
GHRCF/GHRCR
CCRF/CCRR
150 bp

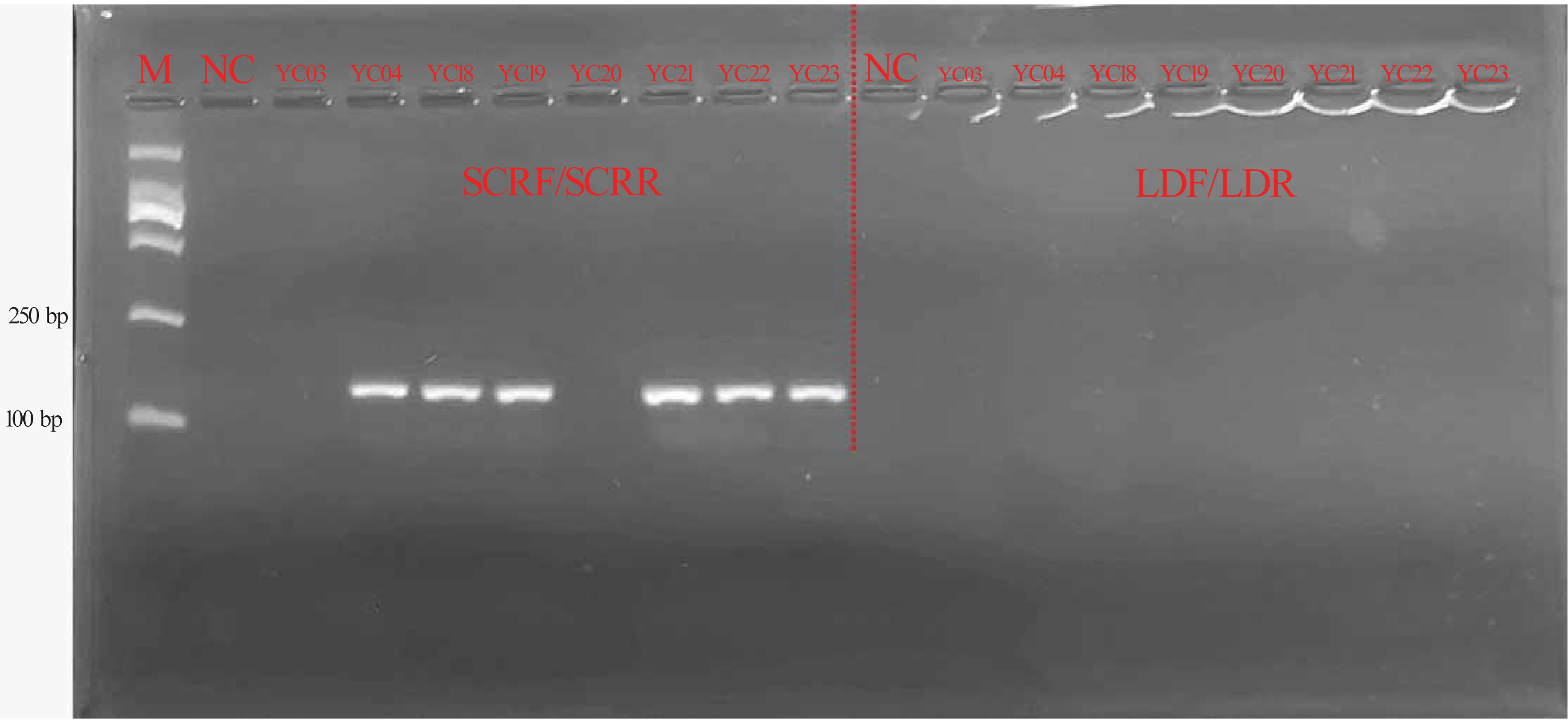
M
NC
YC03
YC04
YC18
YC19
YC20
YC21
YC22
YC23
NC
YC03
YC04
YC18
YC19
YC20
YC21
YC22
YC23
SCRF/SCRR
LDF/LDR
250 bp
100 bp

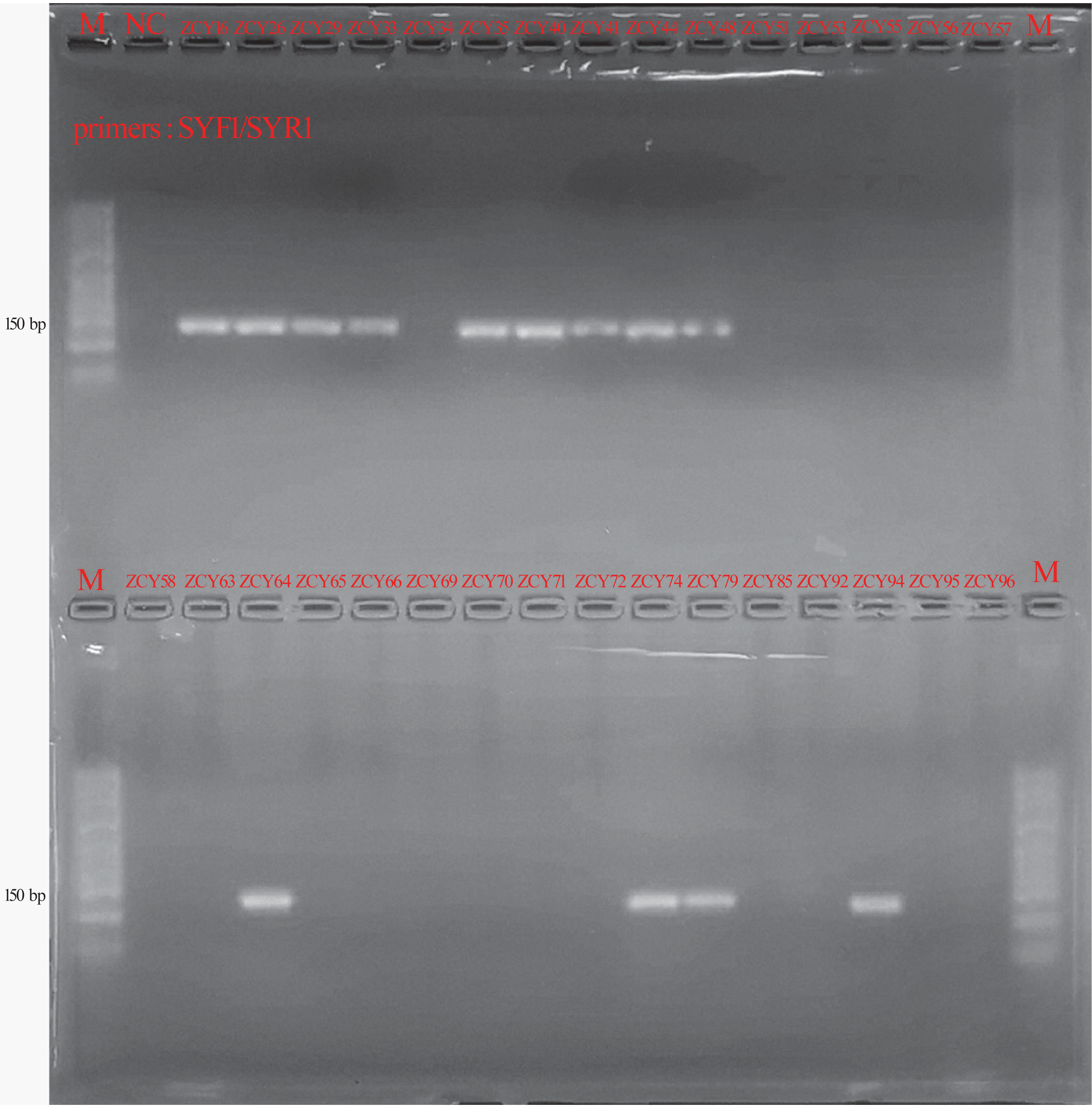
M
NC
ZCY16 ZCY26 ZCY29 ZCY33 ZCY34 ZCY35 ZCY40 ZCY41 ZCY44 ZCY48 ZCY51 ZCY53 ZCY55 ZCY56 ZCY57
M
primers : SYF1/SYR1
150 bp
M
ZCY58 ZCY63 ZCY64 ZCY65 ZCY66 ZCY69 ZCY70 ZCY71 ZCY72 ZCY74 ZCY79 ZCY85 ZCY92 ZCY94 ZCY95 ZCY96
M
150 bp

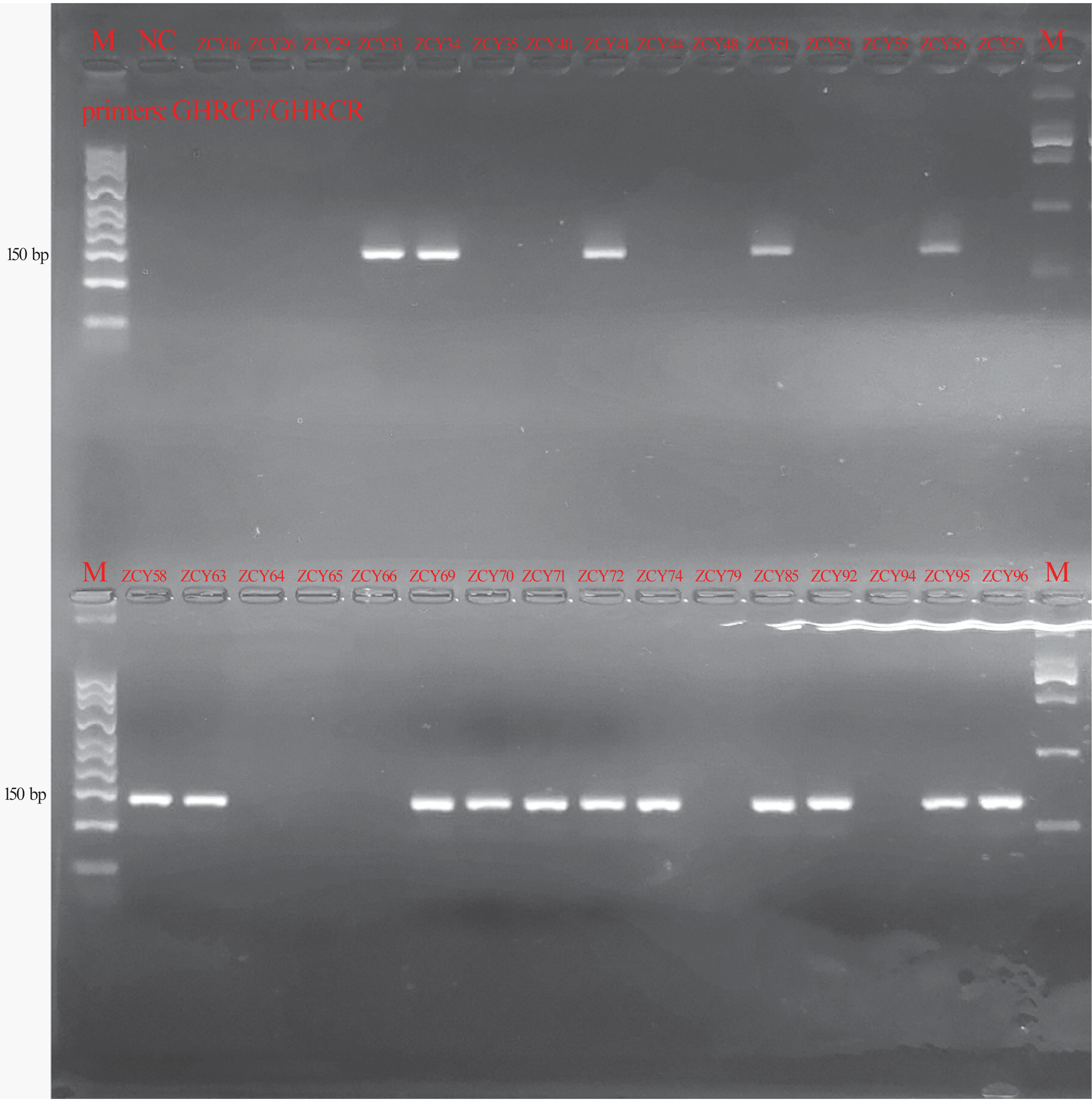
M
NC
ZCY16
ZCY26
ZCY29
ZCY33
ZCY34
ZCY35
ZCY40
ZCY41
ZCY44
ZCY48
ZCY51
ZCY53
ZCY55
ZCY56
ZCY57
M
primers: GHRCF/GHRCR
150 bp
M
ZCY58
ZCY63
ZCY64
ZCY65
ZCY66
ZCY69
ZCY70
ZCY71
ZCY72
ZCY74
ZCY79
ZCY85
ZCY92
ZCY94
ZCY95
ZCY96
M
150 bp

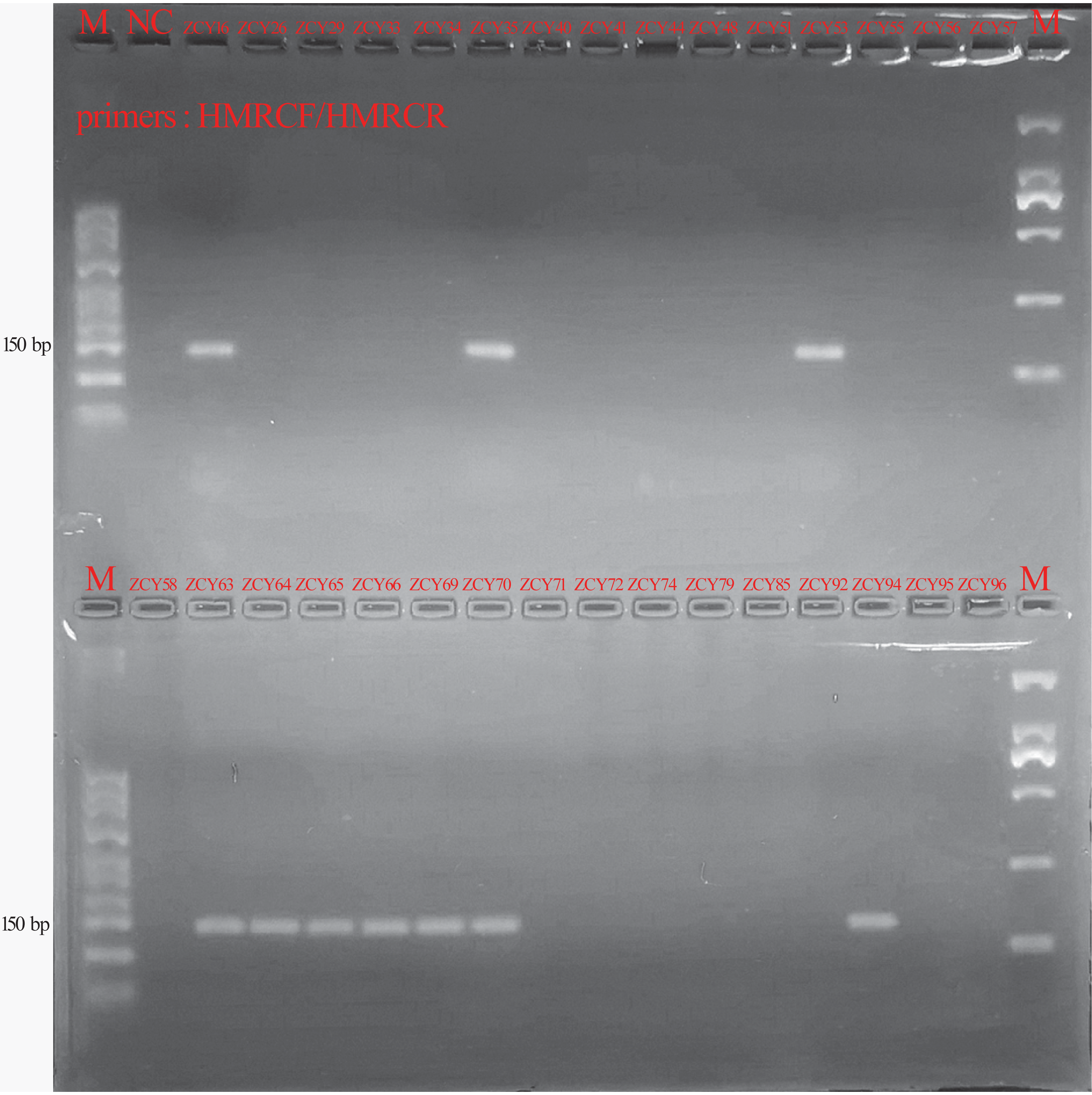
M NC ZCY16 ZCY26 ZCY29 ZCY33 ZCY34 ZCY35 ZCY40 ZCY41 ZCY44 ZCY48 ZCY51 ZCY53 ZCY55 ZCY56 ZCY57 M
primers : HMRCF/HMRCR
150 bp
M ZCY58 ZCY63 ZCY64 ZCY65 ZCY66 ZCY69 ZCY70 ZCY71 ZCY72 ZCY74 ZCY79 ZCY85 ZCY92 ZCY94 ZCY95 ZCY96 M
150 bp

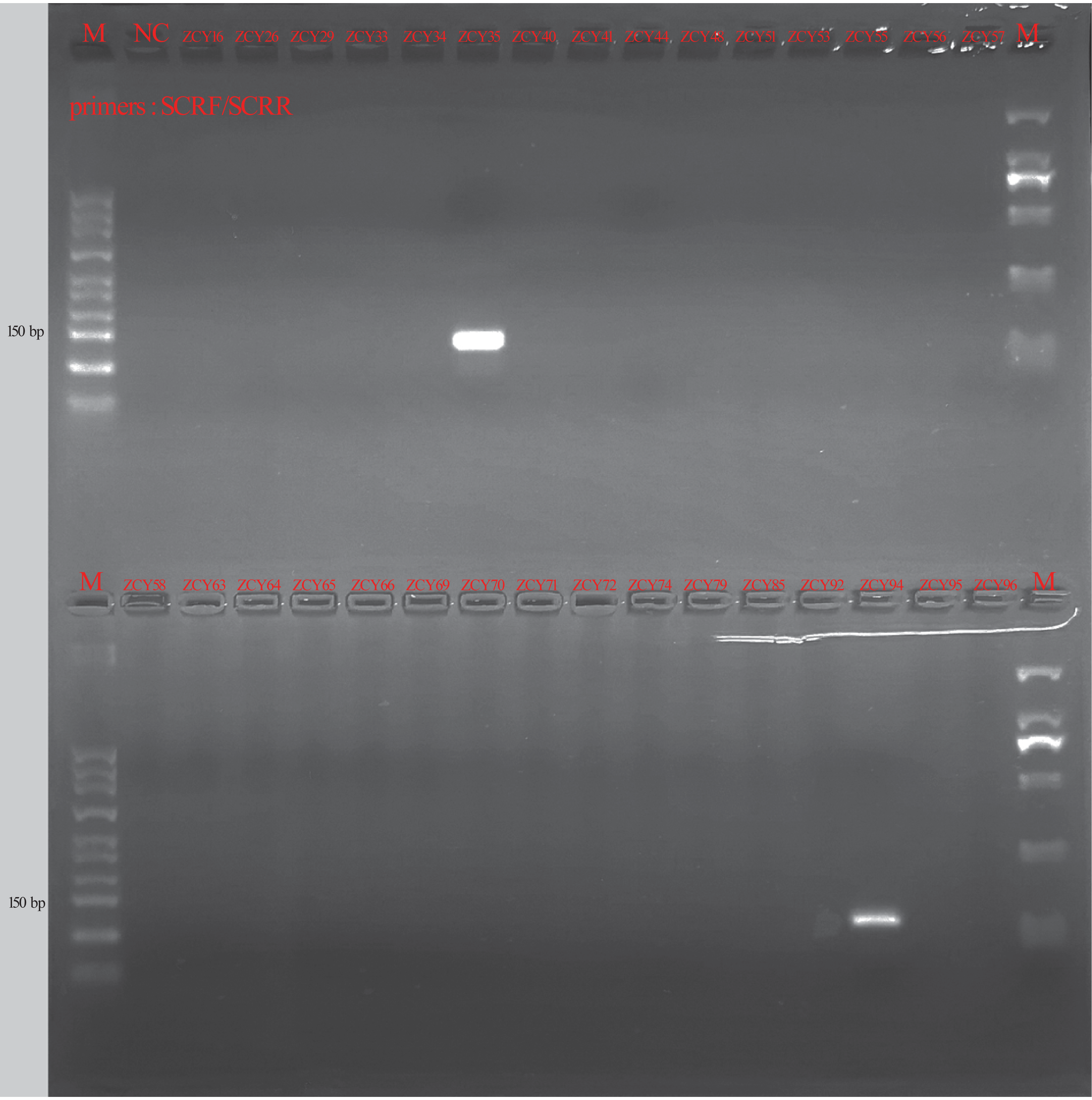
M
NC
ZCY16
ZCY26
ZCY29
ZCY33
ZCY34
ZCY35
ZCY40
ZCY41
ZCY44
ZCY48
ZCY51
ZCY53
ZCY55
ZCY56
ZCY57
M
primers : SCRF/SCRR
150 bp
M
ZCY58
ZCY63
ZCY64
ZCY65
ZCY66
ZCY69
ZCY70
ZCY71
ZCY72
ZCY74
ZCY79
ZCY85
ZCY92
ZCY94
ZCY95
ZCY96
M
150 bp

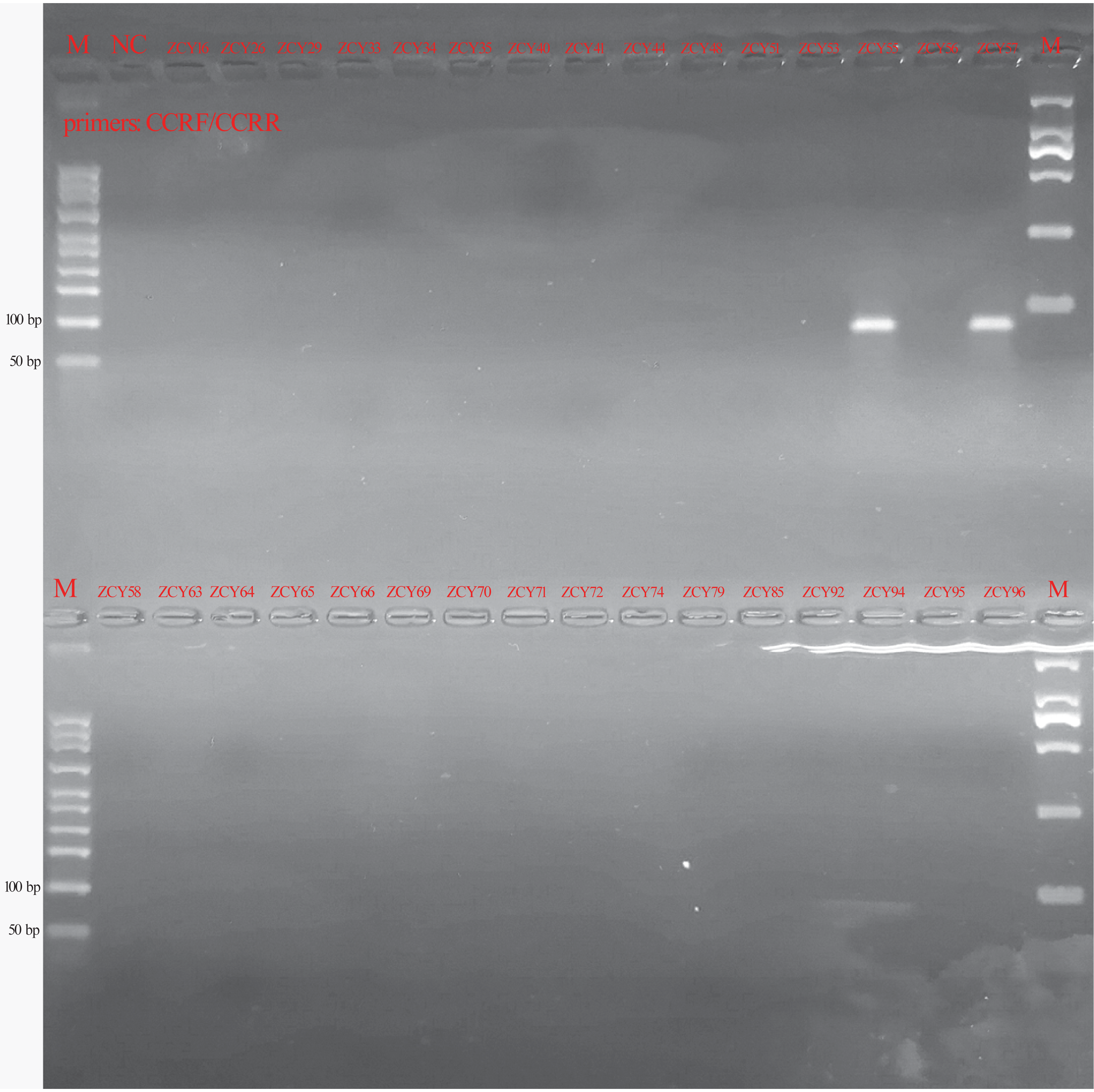
M
NC
ZCY16
ZCY26
ZCY29
ZCY33
ZCY34
ZCY35
ZCY40
ZCY41
ZCY44
ZCY48
ZCY51
ZCY53
ZCY55
ZCY56
ZCY57
M
primers: CCRF/CCRR
100 bp
50 bp
M
ZCY58
ZCY63
ZCY64
ZCY65
ZCY66
ZCY69
ZCY70
ZCY71
ZCY72
ZCY74
ZCY79
ZCY85
ZCY92
ZCY94
ZCY95
ZCY96
M
100 bp
50 bp

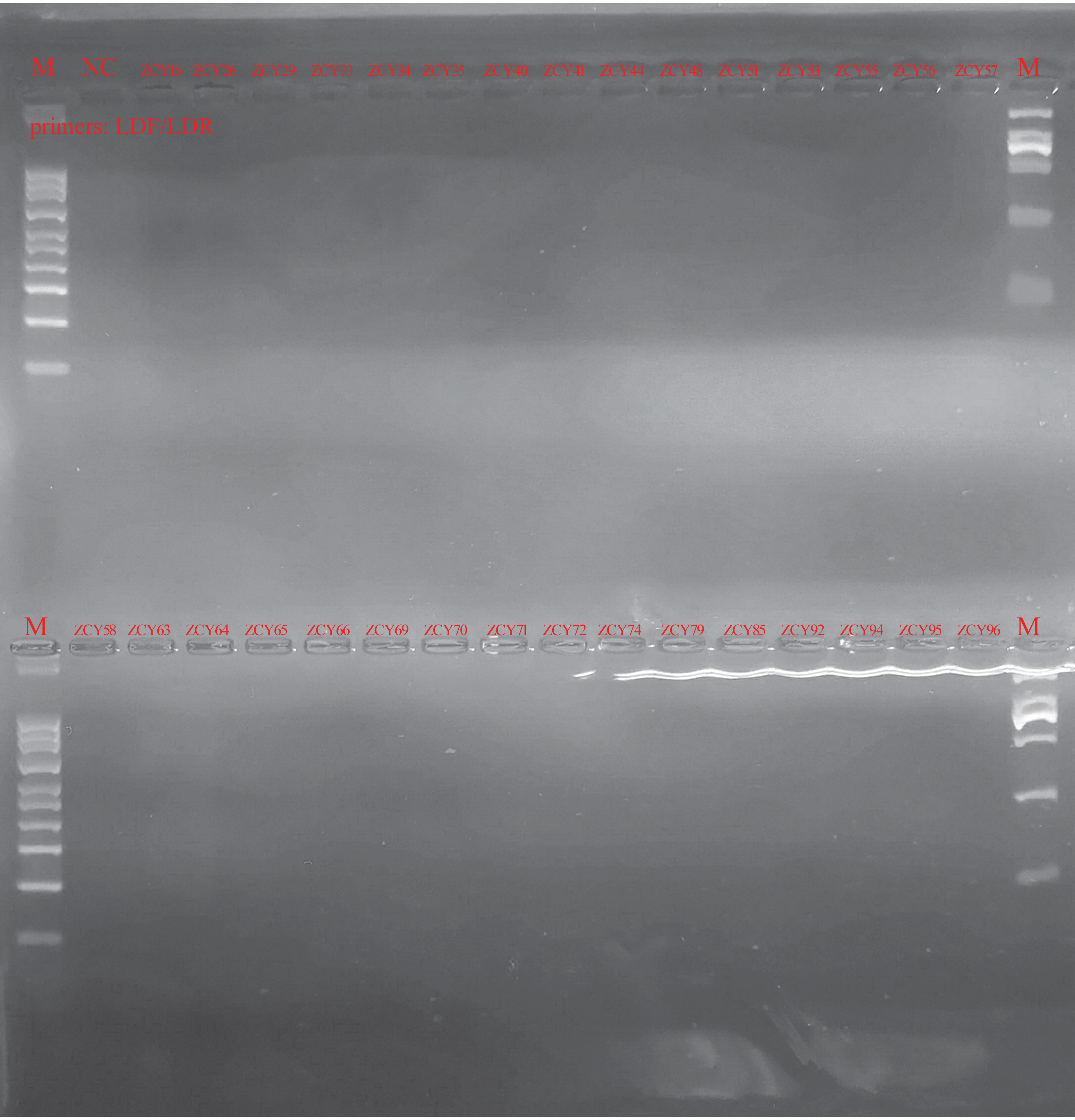
M NC ZCY16 ZCY26 ZCY29 ZCY33 ZCY34 ZCY35 ZCY40 ZCY41 ZCY44 ZCY48 ZCY51 ZCY53 ZCY55 ZCY56 ZCY57 M
primers: LDF/LDR
M ZCY58 ZCY63 ZCY64 ZCY65 ZCY66 ZCY69 ZCY70 ZCY71 ZCY72 ZCY74 ZCY79 ZCY85 ZCY92 ZCY94 ZCY95 ZCY96 M
