# Supplementary material for: Detection of Cistanches Herba (Rou Cong Rong) Medicinal Products Using Species-Specific Nucleotide Signatures
Source: Front Plant Sci. 2018 Nov 13;9:1643. doi: 10.3389/fpls.2018.01643 (PMC6242781; doi:10.3389/fpls.2018.01643)
Supplement: Supplementary Table S3 — The declared compositions of different Chinese patent medicine samples. [file Table_3.DOCX]

Supplementary Table 3 The declared compositions of different Chinese patent medicine samples

| Sample Name | Sample No. | Declared compositions |
| --- | --- | --- |
| Shihu Yeguang pills | ZCY16, ZCY26, ZCY33, ZCY34, ZCY44, ZCY48, ZCY51, ZCY53, ZCY55, ZCY56, ZCY57, ZCY58, ZCY64, ZCY65, ZCY69, ZCY70, ZCY71, ZCY72, ZCY74, ZCY85, ZCY92, ZCY95, ZCY96 | Dendrobii Caulis (*Rou Cong Rong*), Ginseng Radix et Rhizoma (*Ren Shen*), Dioscoreae Rhizoma (*Shan Yao*), Poria (*Fu Ling*), Glycyrrhizae Radix et Rhizoma (*Gan Cao*), Cistanches Herba (*Rou Cong Rong*), Lycii Fructus (*Gou Qi Zi*), Cuscutae Semen (*Tu Si Zi*), Rehmanniae Radix (*Di Huang*), Rehmanniae Radix Praeparata (*Shu Di Huang*), Schisandrae Chinensis Fructus (*Wu Wei Zi*), Asparagi Radix (*Tian Dong*), Ophiopogonis Radix (*Mai Dong*), Armeniacae Semen Amarum (*Ku Xing Ren*), Saposhnikoviae Radix (*Fang Feng*), Chuanxiong Rhizoma (*Chuan Xiong*), Aurantii Fructus (*Zhi Qiao*), Coptidis Rhizoma (*Huang Lian*), Achyranthis Bidentatae Radix (*Niu Xi*), Chrysanthemi Flos (*Ju Hua*), Tribuli Fructus (*Ji Li*), Celosiae Semen (*Qing Xiang Zi*), Cassiae Semen (*Jue Ming Zi*), Powerdered Buffalo Horn Extract (*Shui Niu Jiao Nong Suo Fen*), Goat Cornu (*Shan Yang Jiao*), Honey |
| Wenweishu particles | ZCY29 | Codonopsis Radix (*Dang Shen*), Aconm Lateralis Radix Praeparaia (*Fu Zi*), Astragali Radix (*Huang Qi*), Cinnamomi Cortex (*Rou Gui*), Dioscoreae Rhizoma (*Shan Yao*), Cistanches Herba (*Rou Cong Rong*), Atractylodis Macrocephalae Rhizoma (*Bai Zhu*), Crataegi Fructus, (*Shan Zha*), Mume Fructus (*Wu Mei*), Amomi Fructus (*Sha Ren*), Citri Reticulatae Pericarpium (*Chen Pi*), Psoraleae Fructus (*Bu Gu Zhi*), Dextrin, Sucrose |
| Sanbao capsules | ZCY35, ZCY79, ZCY94 | Ginseng Radix et Rhizoma (*Ren Shen*), Cervi Cornu Pantotrichum (*Lu Rong*), Angelicae Sinensis Radix (*Dang Gui*), Dioscoreae Rhizoma (*Shan Yao*), Testudinis Carapax et Plastrum (*Gui Jia*), Amomi Fructus (*Sha Ren*), Cornifructus (*Shan Zhu Yu*), Ganoderma (*Ling Zhi*), Rehmanniae Radix Praeparata (*Shu Di Huang*), Salviae Miltiorrhizae Radix et Rhizoma (*Dan Shen*), Schisandrae Chinensis Fructus (*Wu Wei Zi*), Cuscutae Semen (*Tu Si Zi*), Cistanches Herba (*Rou Cong Rong*), Polygoni Multiflori Radix (*He Shou Wu*), Chrysanthemi Flos (*Ju Hua*), Moutan Cortex (*Mu Dan Pi*), Paeoniae Radix Rubra (*Chi Shao*), Eucommiae Cortex (*Du Zhong*), Ophiopogonis Radix (*Mai Dong*), Alismatis Rhizoma (*Ze Xie*), Scrophulariae Rad (*Xuan Shen*), Dextrin |
| Kangguzhi Zengsheng pills | ZCY40 | Rehmanniae Radix Praeparata (*Shu Di Huang*), Spatholobi Caulis (*Ji Xue Teng*), Epimedii Folium (*Yin Yang Huo*), Drynariae Rhizoma (*Gu Sui Bu*), Cibotii Rhizoma (*Gou Ji*), Ligustri Lucidi Fructus (*Nu Zhen Zi*), Cistanches Herba (*Rou Cong Rong*), Achyranthis Bidentatae Radix (*Niu Xi*), Raphani Semen (*Lai Fu Zi*), Honey |
| Kanggu Zengsheng pills | ZCY41 | Rehmanniae Radix Praeparata (*Shu Di Huang*), Cistanches Herba (*Rou Cong Rong*), Cibotii Rhizoma (*Gou Ji*), Ligustri Lucidi Fructus (*Nu Zhen Zi*), Epimedii Folium (*Yin Yang Huo*), Spatholobi Caulis (*Ji Xue Teng*), Raphani Semen (*Lai Fu Zi*), Drynariae Rhizoma (*Gu Sui Bu*), Achyranthis Bidentatae Radix (*Niu Xi*), Honey |
| Yucong Qiangshen capsules | ZCY63 | Cornifructus (*Shan Zhu Yu*), Cuscutae Semen (*Tu Si Zi*), Cnidii Fructus (*She Chuang Zi*), Cistanches Herba (*Rou Cong Rong*), Schisandrae Chinensis Fructus (*Wu Wei Zi*), Polygalae Radix (*Yuan Zhi*) |
| Wenweishu capsules | ZCY66 | Codonopsis Radix (*Dang Shen*), Aconm Lateralis Radix Praeparaia (*Fu Zi*), Astragali Radix (*Huang Qi*), Cinnamomi Cortex (*Rou Gui*), Dioscoreae Rhizoma (*Shan Yao*), Cistanches Herba (*Rou Cong Rong*), Atractylodis Macrocephalae Rhizoma (*Bai Zhu*), Crataegi Fructus, (*Shan Zha*), Mume Fructus (*Wu Mei*), Amomi Fructus (*Sha Ren*), Citri Reticulatae Pericarpium (*Chen Pi*), Psoraleae Fructus (*Bu Gu Zhi*), Dextrin, Sucrose |
